# Metabolic Changes During Growth and Reproductive Phases in the Liver of Female Goldfish (Carassius auratus)

**DOI:** 10.3389/fcell.2022.834688

**Published:** 2022-02-28

**Authors:** Claudia Ladisa, Yifei Ma, Hamid R Habibi

**Affiliations:** Department of Biological Sciences, University of Calgary, Calgary, AB, Canada

**Keywords:** seasonal reproductive cycle, targeted metabolomics, LC-MS, chemometrics, growth and reproduction, gonadotropin releasing hormone (GnRH), gonadotropin-inhibitory hormone (GnIH)

## Abstract

Hormones of the brain-pituitary-peripheral axis regulate metabolism, gonadal maturation, and growth in vertebrates. In fish, reproduction requires a significant energy investment to metabolically support the production of hundreds of eggs and billions of sperms in females and males, respectively. This study used an LC-MS-based metabolomics approach to investigate seasonally-related changes in metabolic profile and energy allocation patterns in female goldfish liver. We measured basal metabolic profile in female goldfish at three phases of the reproductive cycle, including 1) Maximum growth period in postovulatory regressed phase, 2) mid recrudescence in fish with developing follicles, and 3) late recrudescence when the ovary contains mature ovulatory follicles. We also investigated changes in the liver metabolism following acute treatments with GnRH and GnIH, known to be involved in controlling reproduction and growth in goldfish. Chemometrics combined with pathway-driven bioinformatics revealed significant changes in the basal and GnRH/GnIH-induced hepatic metabolic profile, indicating that metabolic energy allocation is regulated to support gonadal development and growth at different reproductive cycles. Overall, the findings support the hypothesis that hormonal control of reproduction involves accompanying metabolic changes to energetically support gonadotropic and somatotropic activities in goldfish and other oviparous vertebrates.

## 1 Introduction

A number of oviparous species, including many fishes, are seasonal breeders and follow cycles of predominantly reproductive and growth phases. Integrated control of reproduction, growth and metabolism is multifactorial, involving neurohormones, the pituitary gonadotropins (LH and FSH), growth hormone (GH), gonadal hormones, and thyroid hormones (Zohar et al., 2009; Yaron, 2011; Habibi et al., 2012; Plant, 2015; Ma et al., 2020a; Ma et al., 2020b; Rajeswari et al., 2020; Somoza et al., 2020; Trudeau and Somoza, 2020). Ovarian follicular development starts with the growth of many small early-stage transparent oocytes accumulating neutral lipids in the ooplasm as lipid droplets (early recrudescence). The growing follicles increase in size progressively by taking up glycolipophosphoproteins termed vitellogenin (Vtg) which is the precursor molecule for the yolk proteins during mid recrudescence (Reading and Sullivan, 2011), and eventually form the preovulatory follicles (late recrudescence). A surge of LH and FSH at the end of the ovarian cycle leads to resumption of meiosis, ovulation and spawning, followed by a regressed gonadal phase, which corresponds to a period of maximum growth (Marchant and Peter, 1986; De Leeuw et al., 1989). Changes in metabolism accompany seasonal variation in the reproductive cycle to sustain this energy-demanding process specific to the stage of gonadal development or period of growth. Vitellogenesis requires a significant metabolic energy allocation (Schneider, 2004; Fernandez-Fernandez et al., 2006; Shahjahan et al., 2014; Ladisa et al., 2021) to support the production of hundreds of mature eggs filled with lipoprotein-filled yolk (Jalabert, 2005). This is critical in oviparous species since embryonic development and early larval stages depend entirely on energy molecules stored in the egg during the maturation (Reading and Sullivan, 2011). For example, in rainbow trout, ovaries grow from 0.5% to approximately 20% of the bodyweight prior to ovulation, and during gametogenesis, the oocyte diameter increases from less than 1–5 mm (Tyler et al., 1990). At the end of the vitellogenic period, yolk proteins derived from Vtg contribute to 80–90% of the dry mass of a mature egg (Reading and Sullivan, 2011). Estrogens stimulate the production of Vtg in the liver, which is transported through the bloodstream to the ovaries, and processed into yolk proteins in the oocytes (Jalabert, 2005; Lin et al., 2006; Reading and Sullivan, 2011; Mcbride et al., 2015). In the blood, Vtg also functions as a carrier for various molecules, including calcium, magnesium, iron, vitamins, steroids and thyroid hormones (Reading and Sullivan, 2011). It is well established that dramatic changes occur in the liver of fish related to the gonadal maturation and vitellogenesis (Rinchard and Kestemont, 2003). The reproductive cycle begins, in part, with the production of the hypothalamic gonadotropin-releasing hormone (GnRH) in response to environmental and metabolic cues (Zohar et al., 2009; Plant, 2015). GnRH stimulates the synthesis and release of LH and FSH from the anterior pituitary, promoting gonadal hormones and gametogenesis (Zohar et al., 2009; Yaron, 2011). The reproductive process is integrated with the growth response controlled by GH, which is regulated by a complex network of hormonal and nutritional factors, including GnRH ((Chang et al., 2000; Canosa et al., 2007; Chang and Wong, 2009). All vertebrate species have multiple isoforms of GnRH (Lethimonier et al., 2004; Okubo and Nagahama, 2008; Chang and Pemberton, 2018). The two endogenous GnRH isoforms in goldfish, GnRH-II [chicken GnRH-II] and GnRH-III [salmon GnRH] (Peter et al., 1985; Yu et al., 1988; Kim et al., 1995; Chang and Pemberton, 2018), are involved in the control of pituitary gonadotropin and GH production (Peter et al., 1985; Chang et al., 2000; Klausen et al., 2001) and regulation of seasonal reproductive cycle in goldfish (Omeljaniuk et al., 1989; Gamba and Pralong, 2006; Moussavi et al., 2013; Moussavi et al., 2014; Ma et al., 2020a; Ma et al., 2020b). There is now evidence that gonadotropin-inhibitory hormone (GnIH) is also involved in the multifactorial regulation of growth and reproduction, together with GnRH and thyroid hormones (Ma et al., 2020a; Ma et al., 2020b). In birds and mammals, GnIH inhibits the release of gonadotropins (Tsutsui et al., 2000; Ubuka et al., 2006; Kriegsfeld et al., 2010; Pineda et al., 2010; Tsutsui et al., 2013). However, in other vertebrates like fish, GnIH can exert both inhibitory and stimulatory actions on the production of gonadotropins in a seasonally dependent manner (Amano et al., 2006; Moussavi et al., 2012; Moussavi et al., 2013; Ma et al., 2020a; Ma et al., 2020b). In goldfish, the presence of three GnIH variants has been demonstrated (LPXRFa-1, -2, and-3), although only LPXRFa-3 was identified as a mature peptide (Sawada et al., 2002). In several vertebrates, GnRH and GnIH are also involved in the regulation of growth by modulating GH production (Chang and Wong, 2009). Studies conducted on several fish species, including goldfish, have shown the presence of GnRH receptors on somatotroph cells and demonstrated its stimulatory action on the synthesis and release of GH (Melamed et al., 1995; Klausen et al., 2001; Klausen et al., 2002; Bhandari et al., 2003). Additionally, GnIH can influence growth and metabolism by regulating food intake and GH secretion (Clarke, 2014). GnIH orthologs stimulate GH release in frogs (Koda et al., 2002; Ukena et al., 2003), sockeye salmon (Amano et al., 2006) and rat (Johnson et al., 2007). In goldfish, GnIH regulates GH synthesis and release *in vivo* and *in vitro* in a seasonally-dependent manner (Moussavi et al., 2014; Ma et al., 2020a; Ma et al., 2020b). There is evidence that GnRH receptor level changes between reproductive stages. In goldfish, GnRH receptors undergo seasonal variation, and the highest pituitary content is found during the late recrudescence (Habibi et al., 1989). Similarly, treatments with GnIH and GnRH alone or combined influence pituitary GnIH receptor expression in a seasonally dependent manner (Moussavi et al., 2013; Moussavi et al., 2014). Thus, variation in GnRH and GnIH receptor levels is likely to influence the hormone-mediated seasonal changes. Goldfish undergo a distinct seasonal cycle and provide a suitable model organism to study energy allocation associated with reproduction and growth. We used an LC-MS-based metabolomics approach to investigate the hepatic metabolic profile of adult female goldfish during three stages of the reproductive cycle, including maximum growth period in postovulatory regressed phase, mid recrudescence in fish with developing follicles, and late recrudescence when the ovary contains mature ovulatory follicles. We also investigated seasonally related changes in the liver metabolism following acute treatments with GnRH and GnIH, known to be involved in controlling reproduction and growth in goldfish. Most metabolic pathways and cellular regulatory mechanisms are common among vertebrates, and metabolomics data can be interpreted similarly in different species. However, significant differences in metabolic energy allocation exist when comparing oviparous and viviparous species such as fish and humans, respectively, at different stages of reproduction. The present study provides an insight into the metabolic changes accompanying seasonal variation in the reproductive cycle in fish and other seasonally reproducing vertebrates.

## 2 Materials and Methods

### 2.1 Experimental Animals

In all studies, we used sexually mature (post-pubertal) 4–6 inch (average weigh 34.6 g) goldfish (*Carassius auratus*). Fish were imported by a local supplier (Aquatic Imports, Calgary, AB, Canada) from a fish farm in Pennsylvania (Mercersburg, PA, United States), where they were reared under the natural daylight and temperature cycles. Each experimental group was assigned 20 fish to ensure that a minimum of six female fish could be used in this study. We used fish at three stages of the annual reproductive cycle, including regressed/maximal growth phase (July-August), mid recrudescence (December- January) and late recrudesce (March-April). At arrival, fish were acclimatized for 4–7 days in a flow through system in 25 L tanks kept under the daylight and temperature corresponding to the environmental conditions. Fish were fed a commercial fish diet once a day to satiation (Nutrafin floating pellets; Hagen, Baie d’Urfé, QC, Canada). A buffered MS-222 solution (tricaine methanesulfonate, 160 mg/L, Sigma Aldrich St Louis, MO, United States) was used to anesthetize the fish before intraperitoneal injection and euthanization following the protocols approved by the University of Calgary animal care committee (protocol #AC19-0161) under the guidelines of the Canadian Council of Animal Care. Ovarian stages were assessed by visual inspection after euthanization before tissue collection. Photographs of ovaries used at different gonadal stages are shown in Figure 4. Immediately after collection, liver tissue samples were snap-frozen in liquid nitrogen to arrest the metabolism and stored at −80°C until further analysis.

### 2.2 Hormones and Injection Treatments

GnRH-III (salmon GnRH) (Pyr-HWSYGWLPG-NH_2_) was purchased from Bachem (Torrance, CA, United States). Goldfish GnIH (LPXRFa-3; SGTGLSATLPQRF-NH_2_) was synthesized by the University of Calgary Peptide Services (Calgary, AB, Canada). Before injection, hormones were dissolved in phosphate-buffered saline (PBS). GnRH-III, GnIH, and PBS (control) were administered intraperitoneally twice, at 9 am (T-0 h) and 9 pm (T-12 h; Table 1). Fish were euthanized, and tissue samples were collected 24 h after the final injection. The control group (Group 1) received a sham injection of PBS at T-0 and T-12 h. The injection protocol was chosen based on previous studies demonstrating that double injection with GnRH or GnIH had a more significant stimulatory effect on GH and LH release in goldfish, compared to single injection indicating that the first injection serves as a primer for tissue response (Klausen et al., 2001; Klausen et al., 2002; Moussavi et al., 2012; Klausen et al., 2014; Ma et al., 2020a; Ma et al., 2020b). Doses of GnRH-III and GnIH were chosen based on previous studies conducted on goldfish (Moussavi et al., 2012; Moussavi et al., 2013; Moussavi et al., 2014; Ma et al., 2020a; Ma et al., 2020b). In particular, GnRH-III: 100 ng/g of fish, and GnIH: 50 ng/g of fish were injected based on the weight of fish in the three experimental seasons.

**TABLE 1:** Injection design of GnRH-III (100 ng/g fish wet weight) and gGnIH (50 ng/g fish) treatments, dissolved in PBS solution. Double injection with PBS (Group 1) served as control group. Intraperitoneal injections were administered at T0 and T12 h followed by samples collection at 24 h post first injection.

Group	T0 h	T12 h
1	PBS	PBS
2	PBS	GnRH
3	GnRH	GnRH
4	PBS	GnIH
5	GnIH	GnIH

### 2.3 Extraction of Metabolites and LC-MS

Several preparation protocols have been developed for the extraction of small molecules from tissue samples, and most involve the addition of organic solvents (Li and Bartlett, 2014). In the present study, we used a water-methanol extraction protocol. This process allowed the extraction of polar metabolites while maintaining the biophysical characteristics of the metabolites present in the sample (Wu et al., 2008; Li and Bartlett, 2014). The metabolites extraction protocol involved homogenizing liver samples in a 50% cold water-methanol solution using a bead-beating homogenizer (TissueLyser II, QIAGEN). Samples (∼50 mg) were extracted with methanol [ratio of 1:20 sample (mg): methanol (μl)]. After homogenization, samples were centrifuged at 13,500 rpm for 20 min, and the supernatant was stored at −80°C until further analysis. A Quality control (QC) group was generated by pooling ∼20 mg from five random samples, extracted and analyzed in five statistical replicates together with the investigated groups (Vuckovic, 2012). Metabolite extracts were analyzed using Ultra-High-Performance Liquid Chromatography (UHPLC) mass spectrometry (MS) coupled with a Thermo Fisher Scientific Q-Exactive HF mass spectrometer. A hydrophilic interaction liquid chromatography column (Syncronis HILIC, Thermo Fisher) was used to separate the metabolites. High-resolution full-scan MS data were acquired using negative-mode electrospray ionization, and data were analyzed with MAVEN freeware (Melamud et al., 2010) based on retention times and *m/z* of standards in a targeted profiling approach. MAVEN’s feature detection algorithm extracts peak groups with retention times and *m/z* matching specified standards (Clasquin et al., 2012). Relative quantification of metabolites is estimated based on the intensity of the analyzed peaks. Area top was used to measure peak intensity, representing the average intensity of the top three points of the peak.

### 2.4 Statistical Analysis

We used normalization by median, log transformation and Pareto scaling approach described previously (Katajamaa and Orešič, 2007) to eliminate noise and artifact. To reduce the innate complexity of metabolomics data, multivariate analysis and dimensionality reduction tools such as Principal Component Analysis (PCA) and Partial Least Squared—Discriminant Analysis (PLS-DA) were used to identify significant differences in the metabolic profile of the groups investigated. PCA was performed on treatment groups and the QC group to validate the LC-MS analysis’s accuracy and identify the presence of outliers (Sangster et al., 2006). Moreover, PLS-DA was performed on the treatment groups to highlight differences in metabolic profiles and identify important metabolites (Lee et al., 2018).

*R* ^2^ and *Q* ^2^ parameters estimate the “goodness of fit” and “goodness of prediction” for each model, respectively (Triba et al., 2015). PLS-DA models with *R* ^2^ and *Q* ^2^ > 0.5 were deemed good fitting and good predictability, respectively. Furthermore, a cross-validation process (CV-ANOVA) based on a 7-fold cross-validation permutation testing method was used to assess the significance of the PLS-DA models. Models with a *p*-value <0.05 were considered significant.

We used Variable Importance in Projection (VIP) > 1 as a variable selection method and threshold to identify significant metabolites in the PLS-DA models. Metabolites with VIP score >1 were selected for each model to generate heatmaps and pathway analysis (MetPA; MetaboAnalyst 4.0). The clustered heatmaps display the relative concentration of metabolites and an overview of the metabolic profile for various treatment groups to visualize changes in metabolite concentrations. Hierarchical clustering was conducted on the rows/variables of the data matrix and the dendrograms cluster metabolites that undergo similar changes in abundance. Finally, differences in the levels of metabolites in various groups were tested by one-way ANOVA, followed by Tukey’s post-hoc analyses that identify differences between groups (Table 2). To further investigate differences between seasons, we conducted multiple t-tests, and the *p*-values are also shown in Supplementary Table S5. The effect of single and double injection with GnRH and GnIH on basal metabolism was also analyzed with ANOVA and multiple t-tests (Supplementary Tables S6–S13).

**TABLE 2:** One-way ANOVA results of all the VIP > 1 metabolites identified for the three PLS-DA models investigating the metabolic changes between reproductive phases (Regressed, Mid and Late). Tukey’s post-hoc test was used for multiple comparisons following ANOVA.

VIP metabolites	*p*-value	FDR	Tukey’s HSD
Taurine	1.55 × 10^−5^	0.00041	Late-Regressed; Late-Mid
Glutamic acid	2.16 × 10^−5^	0.00041	Late-Regressed; Late-Mid
GMP	2.36 × 10^−5^	0.00041	Mid-Regressed; Late-Mid
Asparagine	3.75 × 10^−5^	0.00046	Mid-Regressed; Late-Mid
Uridine	4.44 × 10^−5^	0.00046	Mid-Regressed; Late-Mid
Acetylglutamine	0.00011	0.00092	Mid-Regressed; Late-Mid
Ophthalmic acid	0.00019	0.0014	Mid-Regressed; Late-Regressed
Acetoacetic acid	0.00028	0.0017	Late-Regressed; Late-Mid
Uric acid	0.00029	0.0017	Mid-Regressed; Late-Mid
Glucose 1-phosphate	0.0005	0.0026	Late-Regressed; Late-Mid
Adenine	0.0006	0.0026	Mid-Regressed; Late-Mid
Pantothenic acid	0.0008	0.0033	Late-Regressed; Late-Mid
CMP	0.0009	0.0036	Late-Mid
Glutathione	0.0012	0.0044	Late-Regressed; Late-Mid
Glycogen	0.0014	0.0048	Mid-Regressed
Glucose	0.0017	0.0054	Late-Regressed
Inosinic acid	0.0028	0.0081	Mid-Regressed; Late-Mid
Histidine	0.0028	0.0081	Late-Regressed; Late-Mid
Guanosine	0.0058	0.015	Mid-Regressed; Late-Regressed
Adenosine	0.0060	0.015	Mid-Regressed
Fructose	0.0061	0.015	Late-Regressed
Allantoin	0.0078	0.018	Late-Mid
Arginine	0.0081	0.018	Late-Regressed
EAP	0.0088	0.019	Late-Mid
Ornithine	0.011	0.022	Late-Mid
Citrulline	0.011	0.023	Late-Mid
Creatine	0.013	0.024	Late-Regressed; Late-Mid
Aminoadipic acid	0.015	0.028	Mid-Regressed; Late-Mid
AMP	0.016	0.028	Late-Mid
UMP	0.016	0.028	Late-Regressed; Late-Mid
Alanine	0.017	0.029	Late-Regressed; Late-Mid
Valine	0.021	0.032	Late-Regressed
Inosine	0.021	0.032	Mid-Regressed
Tyrosine	0.022	0.032	Late-Regressed
Phenylalanine	0.022	0.032	Late-Regressed
Malonate	0.022	0.032	Late-Mid
Lysine	0.029	0.040	Late-Regressed
Pterin	0.030	0.040	Late-Mid
Leucine	0.030	0.040	Late-Regressed
Ciliatine	0.035	0.045	Late-Regressed

*p*-value <0.05 indicates statistically significant differences between groups. False Discovery Rate (FDR) indicates the *p*-value adjusted for multiple comparisons.

*p*-value adjusted for multiple comparisons using False Discovery Rate (FDR) < 0.05 indicates a significant difference between groups.

### 2.5 Pathway Analysis

The concentration levels of the VIP > 1 metabolites identified for every PLS-DA model were used to conduct pathway analysis and determination of metabolic pathways involved in different treatment groups. In the present study, we used the MetPA metabolomics pathway analysis offered by the Metaboanalyst 4.0 platform as an analytical tool (Xia et al., 2010). Pathway analysis in MetPa combines quantitative enrichment analysis based on the metabolite concentration values and topological analysis that measures the importance of a metabolite in the metabolic pathway. The nodes importance value (pathway impact) is calculated from centrality measures, normalized by the sum of the importance value of metabolites in the pathway (Xia et al., 2010). In this regard, the impact value of zero indicates a relatively low number of connections of one node to others. This suggests that the matched metabolites for a specific pathway have a marginal role concerning the “length” and complexity of the pathway. The global test and Relative-betweenness Centrality were selected as algorithms for the enrichment and topological analysis, respectively (Chong et al., 2019). In the present study, we used the zebrafish (*Danio rerio*) KEGG pathway library as a reference.

## 3 Results

### 3.1 Characterization of Fish Hepatic Metabolism at Different Stages of the Seasonal Cycle

**FIGURE 1:**
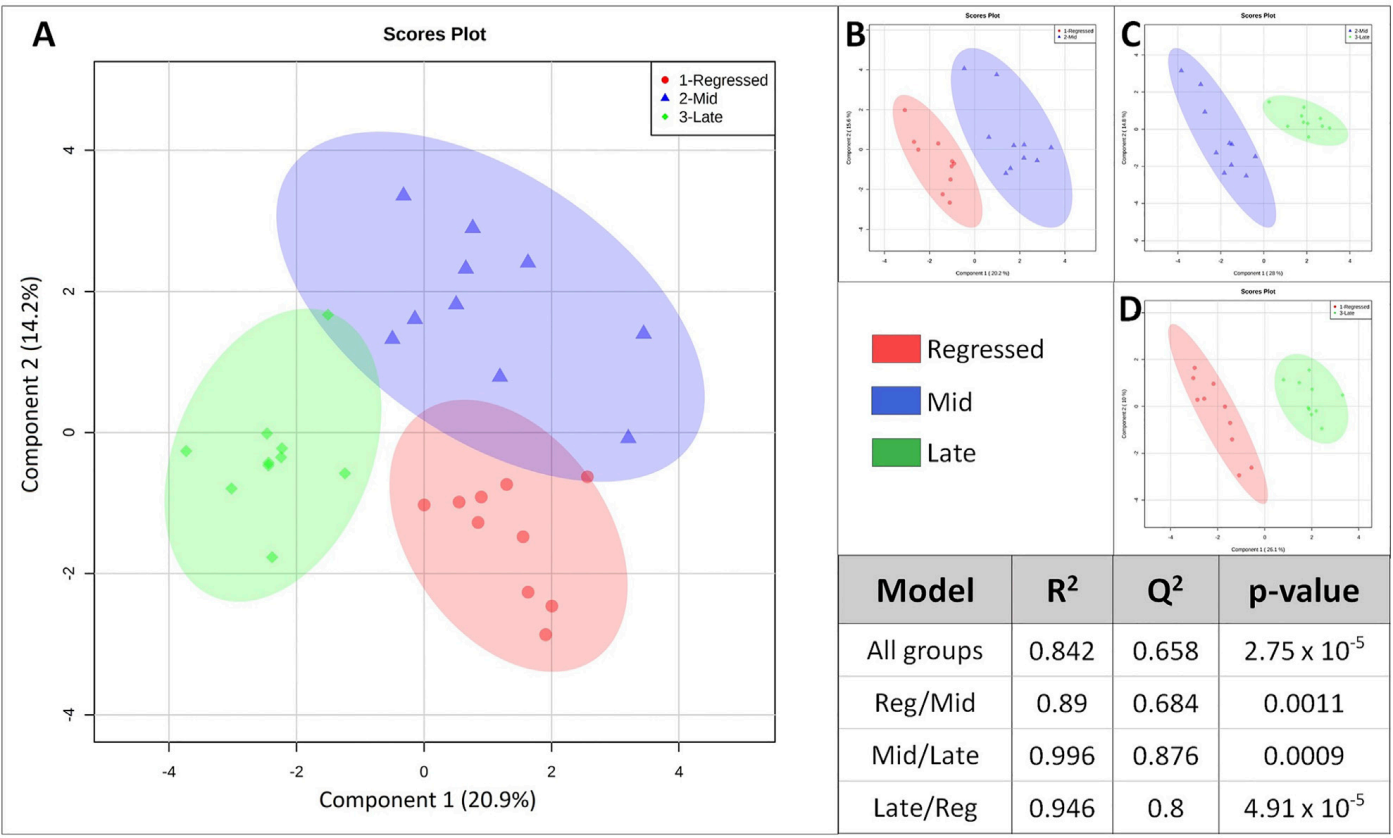
Score scatter plot of the built Partial-Least Squares—Discriminant analysis (PLS-DA) models. The “All groups” model investigate the change in liver metabolism of the three groups corresponding to the three reproductive stages **(A)**. Groups were also analyzed in a pair-wise manner: Regressed/Mid **(B)**, Mid/Late **(C)** and Late/Regressed **(D)**. Each axis represents a component that is a source of variation between samples (shown as percentage in parenthesis). Each point represents normalized concentrations of the complete metabolite set of a liver sample. Regressed: regressed gonadal phase/somatotropic phase (n:10); Mid: mid gonadal recrudescence (n:10); Late: late gonadal recrudescence (n:10). The table shows results for statistical analysis obtained for the built models. R^2^ assess the “goodness of fit” and *Q* ^2^ asses the “goodness of prediction”. *p*-values were measured *via* CV-ANOVA based on a 7-fold cross validation permutation testing method. *p*-value <0.05 indicate significant difference.

**FIGURE 2:**
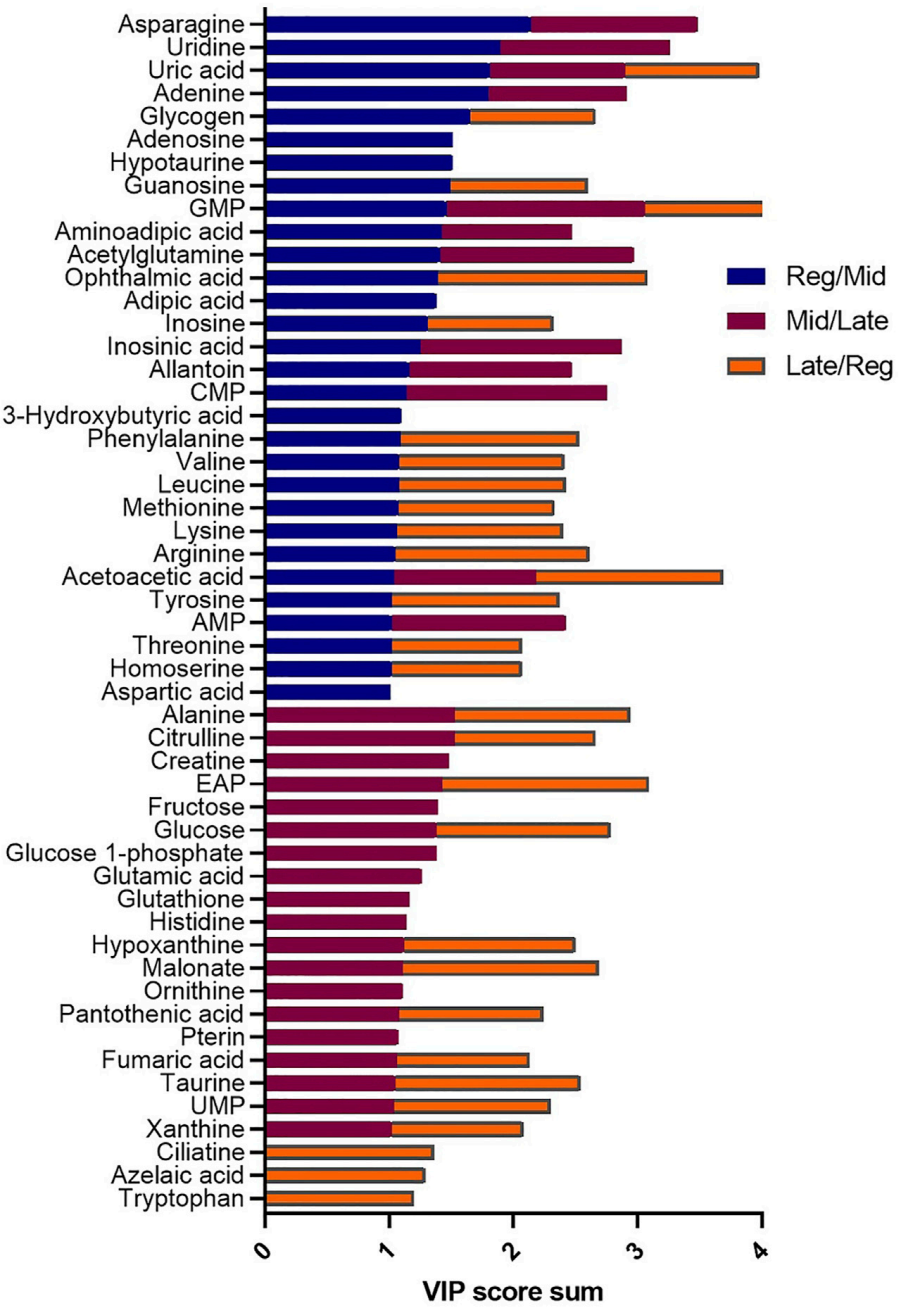
Stacked bar graph comparing the VIP > 1 metabolites the three PLS-DA models: Reg/Mid (blue), Mid/Late (burgundy), Late/Reg (orange). Colored bars represent the VIP score (>1) in each model as contribution to the whole. The VIP score estimates the importance of each metabolite in the PLS-DA models providing useful insight on the differences between models.

**FIGURE 3:**
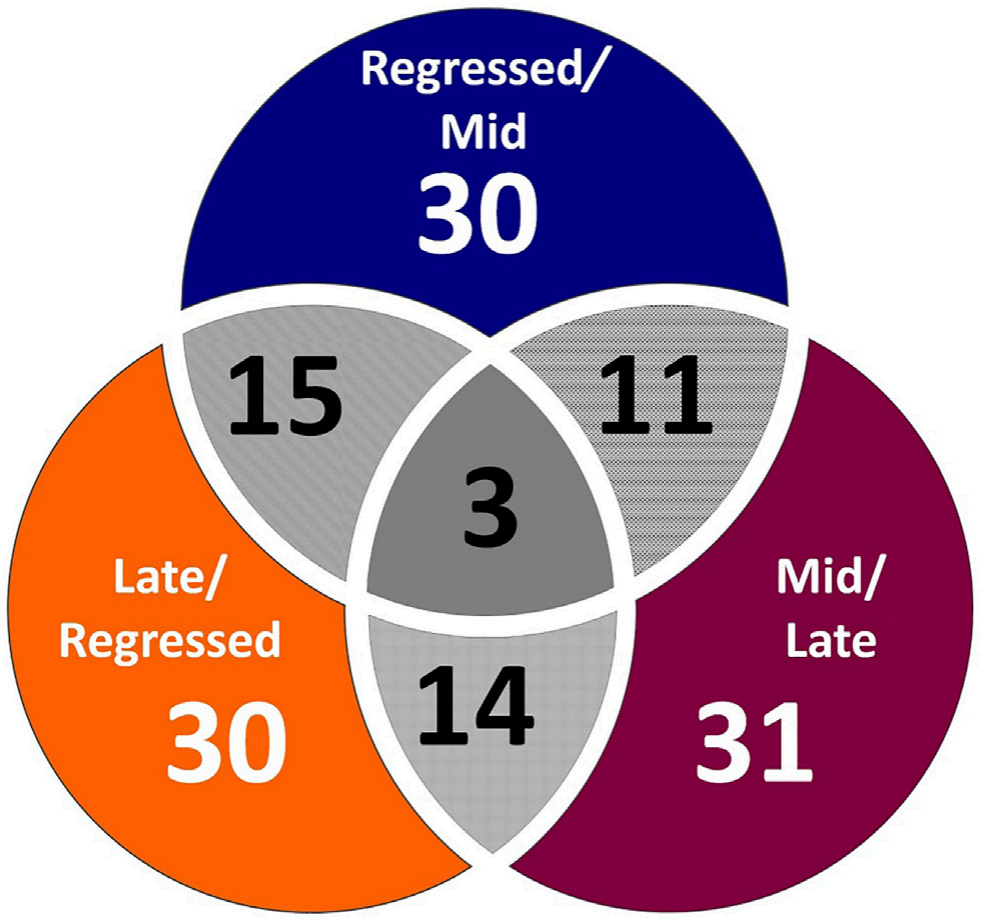
Venn diagram displaying the number of unique and common VIP > 1 metabolites between PLS-DA models. The Regressed/Mid PLS-DA model has 30 metabolites with VIP score >1 and share 15 metabolites with the Late/Regressed model and 11 with the Mid/Late model. The Late/Regressed and the Mid/Late model have 30 and 31 VIP > 1 metabolites, respectively and 14 are in common between the two models. Three metabolites are shared by the three models: Acetoacetic acid, Guanosine monophosphate (GMP) and Uric acid.

**FIGURE 4:**
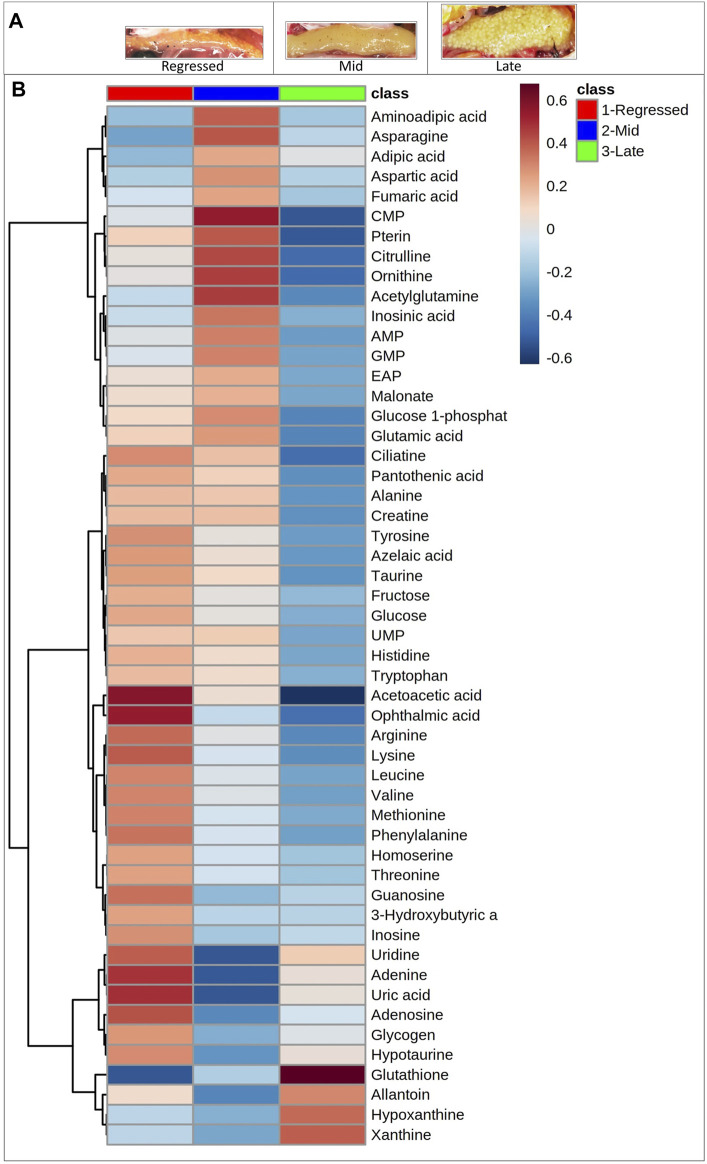
Photographs of ovaries used at different gonadal stages **(A)**. Clustered heatmap of the VIP > 1 metabolites identified in the three models. Shades of blue and red indicate decreases and increases in the concentrations of metabolites in each reproductive phase, respectively **(B)**.

**FIGURE 5:**
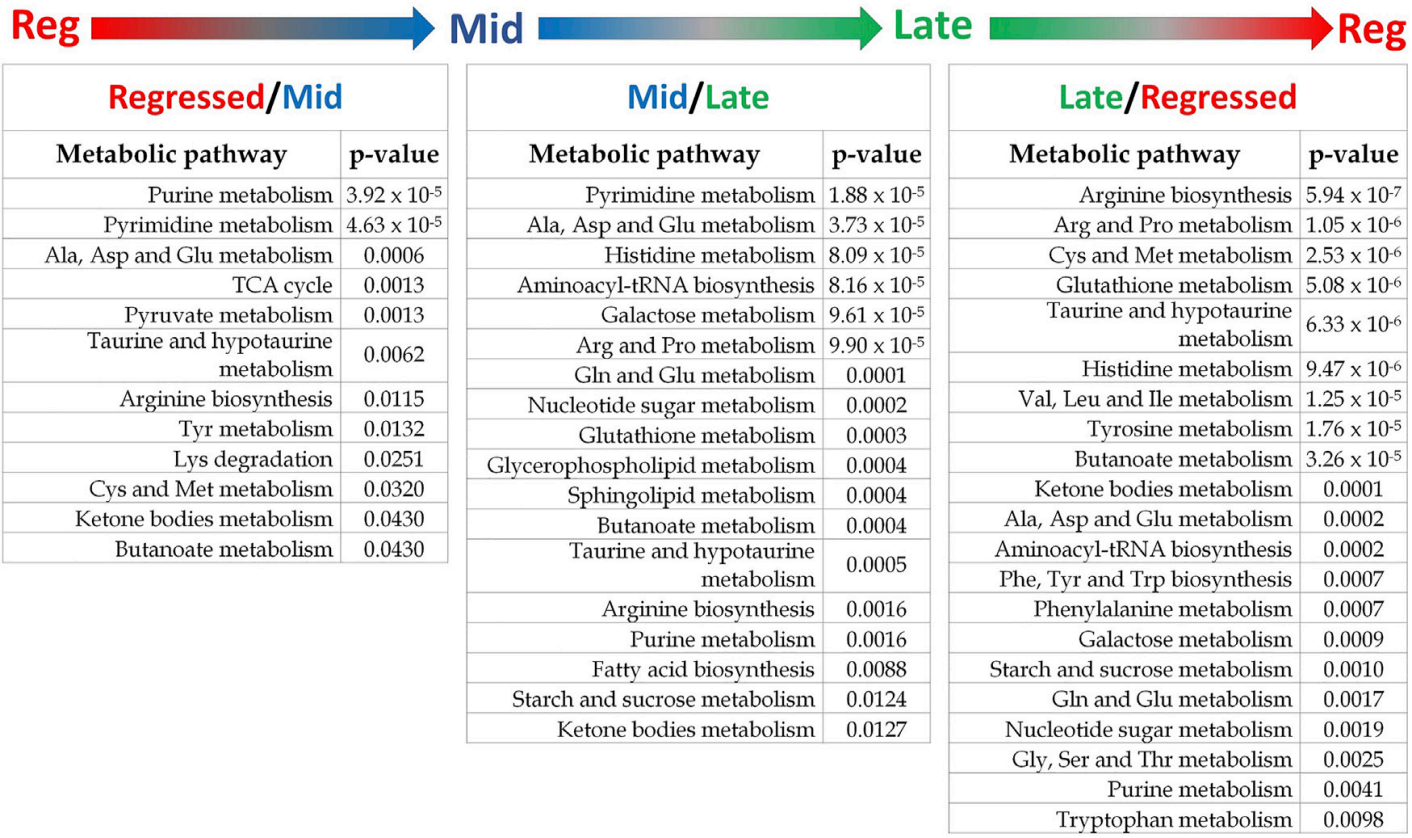
Results summary of pathway analysis showing the main altered metabolic pathways in each comparison. Pathways with *p*-value < 0.05 are considered significantly altered between the investigated phases. The detailed results of pathway analysis for each comparison are shown in Supplementary Tables S2–S4.

Female liver samples collected at different stages of the reproductive cycle were analyzed using LC-MS for relative quantification of metabolites. Samples were analyzed using negative-mode electrospray ionization, and complementary analysis using positive-mode was not carried out in the present study. A targeted metabolomics profiling approach using an *m/z* and retention times standard library led to the identification of 71 metabolites in the aqueous phase of liver extracts. Examples of peak detection and measurements are shown in Supplementary Figure S1. Furthermore, the average peak intensity (±SEM) of the VIP > 1 metabolites during the three investigated reproductive stages is provided in Supplementary Table S1. In this study, we used both univariate and multivariate statistical and visualization techniques to comprehensively characterize the metabolic profile of different reproductive stages and investigate seasonal variations in metabolism. Chemometrics tools such as PLS-DA and PCA were used to reduce the dimensionality of the dataset and to visualize similarities (cluster formation) and dissimilarities (cluster separation) between the investigated groups. PCA was conducted on the metabolic data obtained from the Control samples collected from female goldfish at three stages of reproduction. The three groups included fish at regressed gonadal phase/somatotropic phase (*n* = 10), mid recrudescence (*n* = 10), and late recrudescence (*n* = 10). We also used a quality control group (QC, *n* = 5) (Supplementary Figure S2). The PCA scatter plot shows a strong cluster formation for the QC group, confirming the reliability of our dataset and the absence of outliers as all samples fall within the 95% confidence area. We performed PLS-DA on the dataset to further explore the shift seen in the PCA analysis. In the supervised multivariate modelling method, the PLS-DA algorithm combines dimensionality reduction and discriminant analysis, using the group ID of samples. Hence, PLS-DA provides a robust predictive and descriptive regression model (Lee et al., 2018). The score scatter plot obtained from PLS-DA analysis revealed a significant shift in the metabolic profile of the three gonadal stages (Regressed, Mid and Late; Figure 1). Statistical significance of the identified shift was assessed by the *R* ^2^ and *Q* ^2^ parameters (*R* ^2^ = 0.842, *Q* ^2^ = 0.658) and by CV-ANOVA (*p* = 2.75 × 10^−5^) (Figure 1). To further investigate the observed metabolic shift, we performed pairwise analysis for each group and obtained significant differences for all PLS-DA models: Regressed/Mid: 0.0011; Mid/Late: 0.0009; Late/Regressed: 4.91 × 10^−5^ (Figure 1). In the present study, each season was compared with the other two. We identified the dominant metabolites in each PLS-DA model by selecting the metabolites with VIP score >1 to characterize the phenotypes at the three recrudescence stages investigated (Figure 2). Given the large number of variables provided by LC-MS analysis, data sets are often difficult to interpret and feature selection prior to pattern recognition becomes a critical step in the metabolomics studies (Lê Cao et al., 2011). Thus, metabolites with a VIP score >1 can be regarded as molecular markers that indicate the status of a given condition and differences among the treatment groups (Gorrochategui et al., 2016). The stacked bar graph shown in Figure 2 compares the VIP score (>1) of the identified metabolites in each model as a contribution to the whole dataset (Figure 2). Furthermore, Figure 2 shows similarities between models and highlights differences in the contribution of variables, suggesting that the hepatic metabolic profile of the three stages of reproduction is considerably different. Figure 3 illustrates the relationship between the three built models by showing the number of overlapping variables. The Venn diagram can directly compare models, determine the number of identified VIP > 1 metabolites in each model, and highlight the number of unique and shared variables. In the Regressed/Mid models, we identified 30 VIP > 1 metabolites where 15 metabolites were similar to the Late/Regressed model and 11 with the Mid/Late model. We also identified 30 metabolites with VIP > 1 in the Late/Regressed model and 31 in the Mid/Late-model, with 14 common metabolites between these two. The three metabolites with a VIP score >1 common in all groups were Acetoacetic acid, Guanosine monophosphate (GMP) and Uric acid. We combined the metabolites with VIP > 1 of each model for heatmap generation among the 52 distinguishing metabolites. The clustered heatmap presented in Figure 4 shows the group-average concentration of all VIP > 1 metabolites, providing insight into the relationship between metabolites in the three reproductive stages, facilitating the visualization of their relative abundance (Figure 4). Additionally, the hierarchical clustering analysis accompanying the heatmap revealed the presence of three primary metabolites clusters since three main branches occur at similar distances (Figure 4). The interpretation of the heatmap is further facilitated by one-way ANOVA results (Table 2) and multiple t-test comparisons (Supplementary Table S5). Finally, to fully characterize the changes in basal metabolism related to the three reproductive stages, we conducted a pairwise pathways analysis to investigate the differences. Metabolites with VIP > 1 obtained in the three built models were submitted to the Metaboanalyst 4.0 platform for the pathway analysis (Xia et al., 2010). The analysis of the biochemical processes data in this study was based on the zebrafish (Danio rerio) pathway library of the Kyoto Encyclopedia of Genes and Genomes (KEGG) (Kanehisa et al., 2019). Pathway analysis used in this study integrates quantitative pathway enrichment analysis (QEA) that measures differences in metabolites concentration between groups (*p*-value), and network topology analysis that estimates the relative importance of metabolites based on their relative position in a given pathway (pathway impact). The results of the pairwise pathway analysis shown in Figure 5 describe differences in metabolic activities and patterns of energy allocation between the seasons. The detailed results of the pathways impacted in each comparison are presented in Supplementary Tables S2–S4. Our results demonstrate that purine and pyrimidine metabolism are among the main pathways contributing to the differences observed between the fish at regressed phase and mid recrudescence, and between mid and late recrudescence groups (Figure 5). Changes in the concentration of metabolites such as guanosine, inosine, adenine, and adenosine suggest increased activity of the purine salvage pathway during the regressed phase compared to mid recrudescence (Figure 4). On the other hand, the high levels of inosinic acid, AMP and GMP suggest an increased *de novo* purine synthesis during mid recrudescence, compared to regressed and late recrudescence (Figure 4). On the contrary, elevated concentration of allantoin, hypoxanthine and xanthine in late compared to mid recrudescence suggests an enhanced purine degradation in the late recrudescence stage (Figure 4). Pathway analysis comparing regressed and mid recrudescence and regressed and late recrudescence indicate changes in the metabolism of several amino acids, including taurine, arginine, tyrosine, lysine, methionine, valine, leucine, histidine, alanine, threonine and phenylalanine (Figure 5). The heatmap, supported by ANOVA and multiple t-test analysis (Table 2; Supplementary Table S5), indicates that these amino acids have significantly higher concentrations during the regressed phase than mid and late recrudescence. Thus, the results suggest higher levels of amino acid turnover to support protein synthesis during the stage of maximal growth (gonadal regressed phase; Figure 4). The glycogen concentration was significantly higher in the regressed than the mid and late recrudescence groups, as shown in the heatmap (Figure 4) and the ANOVA and t-test analysis (Table 2; Supplementary Table S5). In fish, the liver is the main site for glycogen buildup, and lower concentrations occur in the brain and the skeletal muscle (Soengas et al., 1993). Our results demonstrate an enhanced glycogen synthesis during the regressed phase, consistent with previous studies in brackish-water fish, common roach *R. rutilus* (Rinchard and Kestemont, 2003) and flounder (Petersen and Emmersen, 1977). Finally, taurine, hypotaurine and tyrosine metabolism are enhanced during the regressed phase, as shown in the heatmap and pathway analysis comparing regressed to mid and late recrudescence (Figures 4, 5). The functional amino acid taurine and thyroid hormones (derived from tyrosine) are essential for normal growth (Power et al., 2001; Andersen et al., 2016). The results demonstrate enhanced anabolic pathways such as protein and glycogen synthesis during the regressed gonadal phase, corresponding to the stage of the maximal growth period. Pathway analysis comparing mid and late recrudescence metabolic profiles demonstrate significant differences in lipid metabolism (Figure 5). Based on the results of distinguishing VIP metabolites and enriched pathways, fatty acid biosynthesis, sphingolipid, and glycerophospholipid metabolism were more active in the liver of mid recrudescence fish than late (Figure 4). This is corroborated by the ANOVA results of *O*-Phosphoethanolamine (a metabolite of the phospholipid synthesis pathway; EAP in the heatmap) and malonate (first step product of fatty acids synthesis) that indicate a significant increase of these metabolites in mid compared to late recrudescence (Table 2). Consistent with previous studies, these results indicate an enhanced lipid production and mobilization in the liver supporting the vitellogenesis process during mid recrudescence (Rinchard and Kestemont, 2003). Validated by the ANOVA results, mid recrudescence is characterized by high levels of ornithine and citrulline, metabolites derived from arginine metabolism (Table 2). Results of pathway analysis confirm the different activity of the arginine biosynthesis pathway when comparing regressed and mid recrudescence fish (Figure 5). Arginine metabolism is associated with the reproductive system and can affect fecundity by regulating vitellogenesis and egg quality (Waters et al., 1992). Pathway analysis comparing Mid and late recrudescence highlights changes in the metabolism of several amino acids, including arginine, cysteine, methionine, taurine, hypotaurine, histidine, leucine, tyrosine, phenylalanine, and glutamine (Figure 5). The heatmap in Figure 4 demonstrates lower concentrations of these and other amino acids during the late recrudescence than regressed and mid, suggesting a lower activity of amino acids and protein metabolism in the final stages of gonadal recrudescence. The late recrudescence is also characterized by significantly lower levels of the ketone bodies acetoacetic acid and hydroxybutyric acid compared to regressed and mid recrudescence as shown in the heatmap and ANOVA analysis (Figure 4; Table 2). In male fish, however, late recrudesce was characterized by significantly higher levels of ketone bodies than those at regressed and mid recrudescence (Ladisa et al., 2021). Furthermore, late recrudescence is characterized by significantly higher glutathione levels (Figure 4; Table 2), indicating an enhanced glutathione metabolism as demonstrated by the pathway analysis results comparing late to both regressed and mid recrudescence (Figure 5).

### 3.2 Effects of Acute Treatments With Gonadotropin-Releasing Hormone and Gonadotropin-Inhibitory Hormone on Metabolism

**FIGURE 6:**
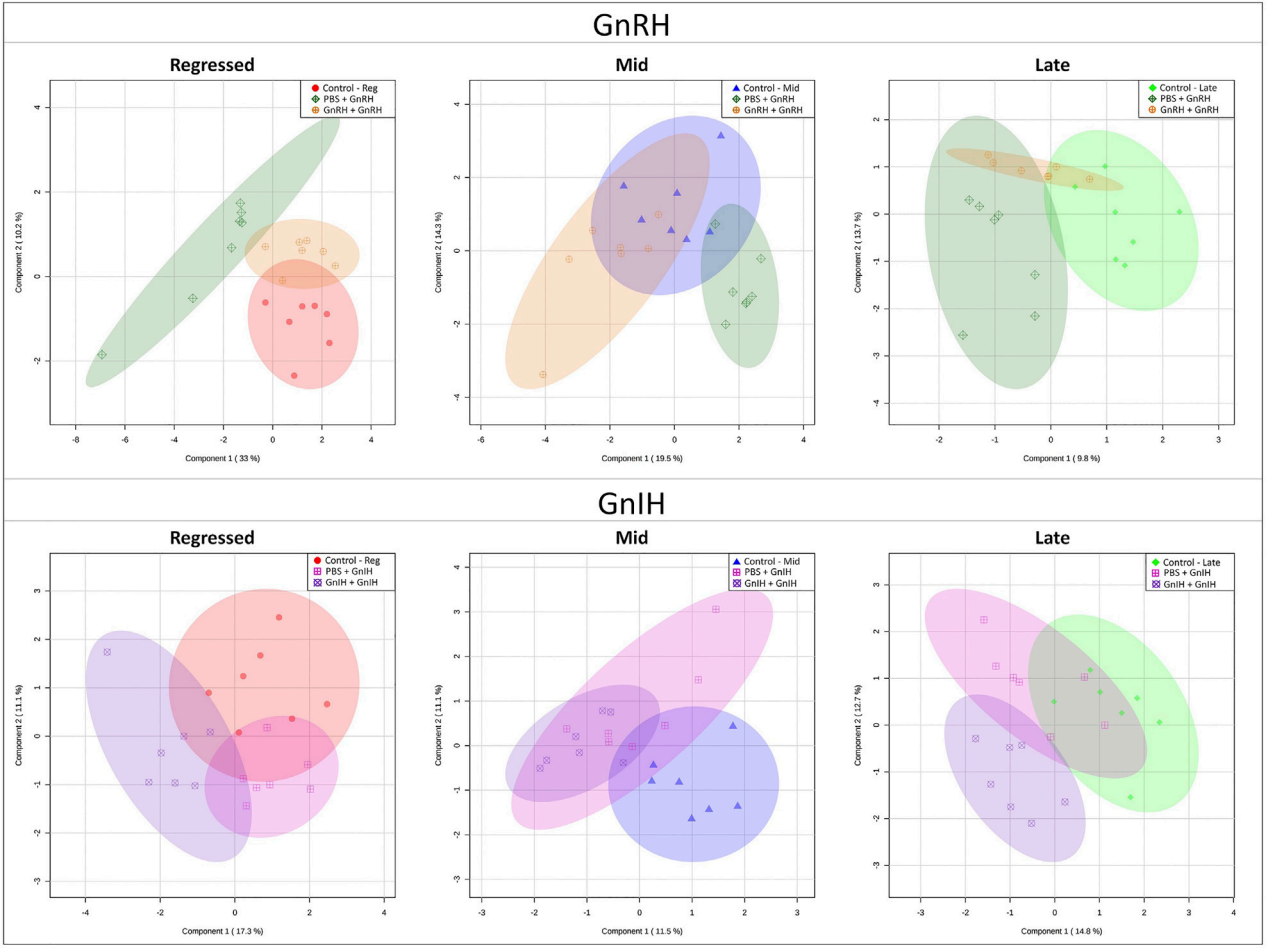
PLS-DA score scatter plots of the models investigating the acute effect of GnRH and GnIH in the three investigated reproductive phases: regressed phase/growth phase, mid recrudescence and late recrudescence. Each graph shows the shift in liver metabolism between Control (PBS + PBS), single injection with GnRH or GnIH (PBS + GnRH/GnIH) and double injection with GnRH or GnIH (GnRH/GnIH + GnRH/GnIH). Each point represents concentrations of the complete metabolite set of a liver sample of the respective group. Axes represent the first and second component that is a source of variation between samples.

**FIGURE 7:**
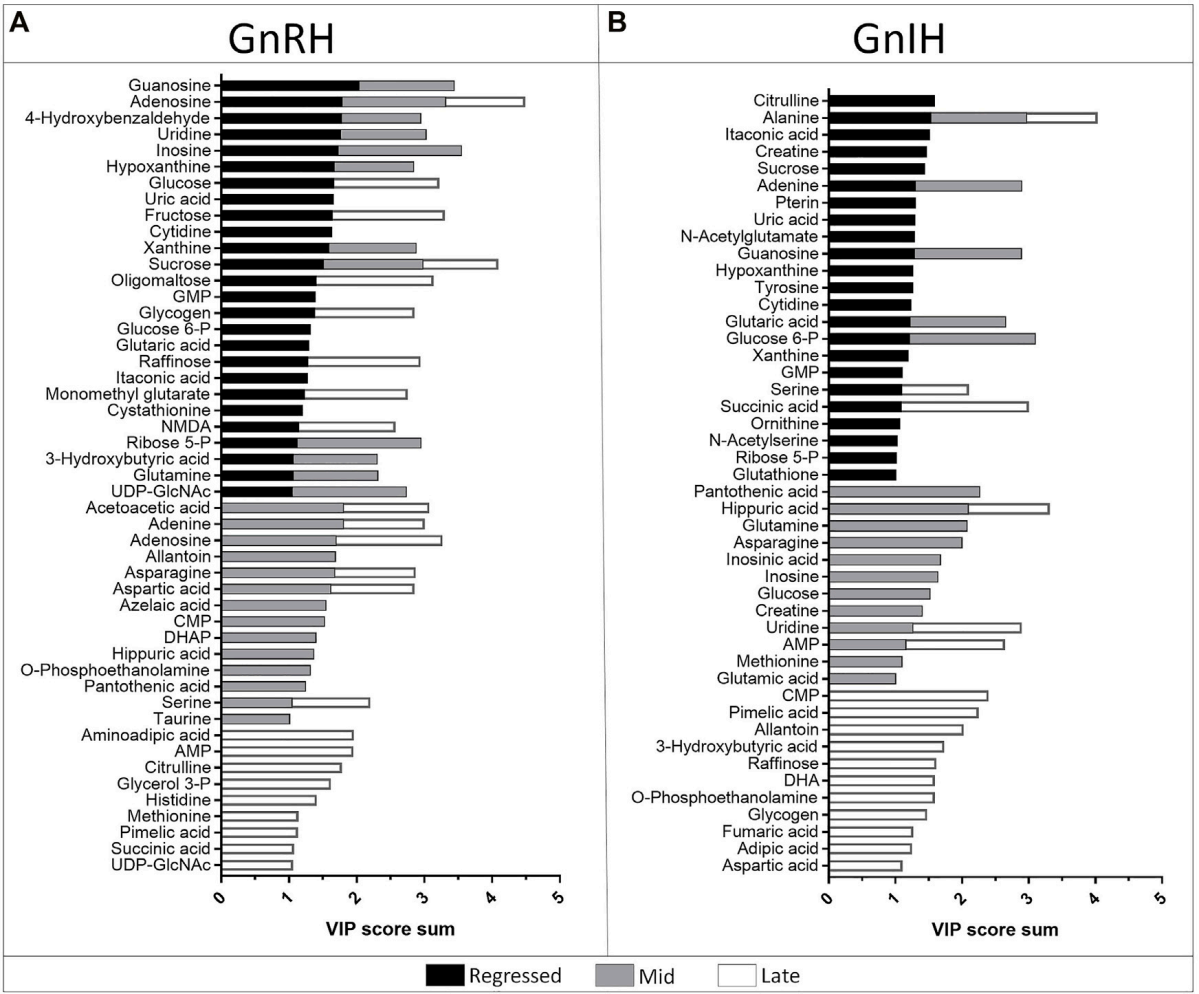
Stacked bar graph showing the metabolites with VIP score >1 identified in the PLS-DA models of GnRH **(A)** and GnIH **(B)** treatments in the three investigated reproductive phases: regressed phase/growth phase (black), mid recrudescence (grey) and late recrudescence (white). Colored bars represent the VIP score of the metabolite as contribution to the whole. For each reproductive phase, the model represents the comparison between three groups: control, single and double injection with GnRH or GnIH.

**FIGURE 8:**
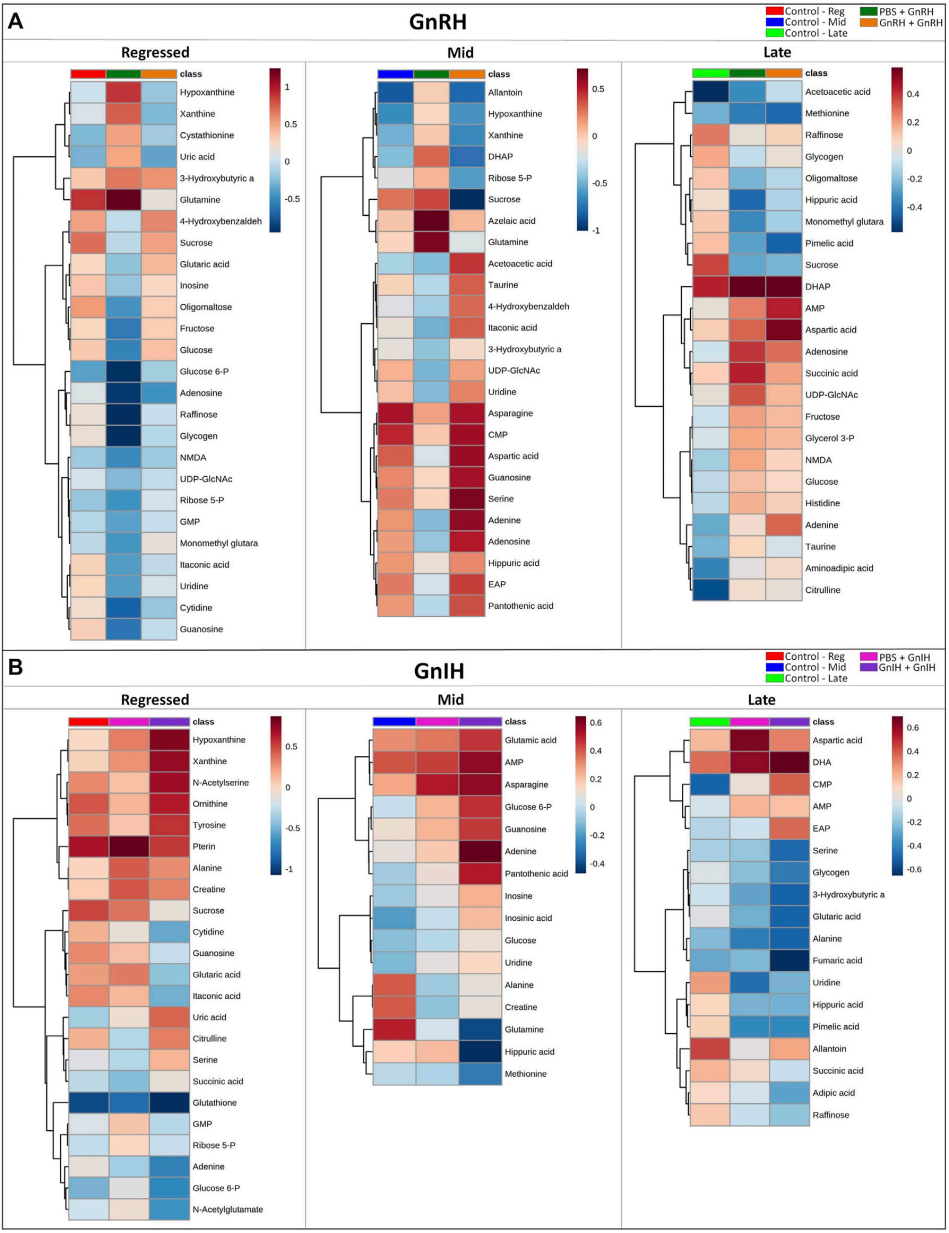
Clustered heatmap showing the metabolites with VIP score >1 of the PLS-DA models for GnRH **(A)** and GnIH **(B)** treatments in the three investigated reproductive phases (Regressed, Mid and Late). For each reproductive phase, the effect of hormones on metabolites was investigated comparing the corresponding control group, single and double injection with GnRH or GnIH groups. Each column represents the group-average concentration of all VIP > 1 metabolites. Decrease or increase of metabolites concentration is indicated by shades of blue and red, respectively. The heatmap was constructed using Ward’s algorithm and the accompanying clustering analysis measures Euclidean distance.

GnRH and GnIH can regulate both gonadotropic and somatotropic activities and are involved in the multifactorial regulation of growth and reproduction in goldfish. We injected GnRH and GnIH in adult female goldfish at three stages of the gonadal cycle to investigate their potential effects on liver metabolism. GnRH-III, one of the two native isoforms in goldfish (Yu et al., 1991), and goldfish GnIH (gGnIH) were used in the present study. Fish received a single or double injection of GnRH-III or GnIH at T0 and T12 h and sacrificed 24 h after the first injection, as shown in Table 1. Injections with PBS (PBS + PBS) served as the control (Ctrl) in the present study. The time-course of the treatments was based on previous studies with GnRH on goldfish in which the first injection served as a priming treatment. Multiple GnRH injections were shown to enhance subsequent LH production (Omeljaniuk et al., 1989; Moussavi et al., 2012, Moussavi et al., 2013, Moussavi et al., 2014). However, it is likely that the acute nature of GnRH and GnIH treatments used in the present study may not be sufficient to elicit changes seen in different seasons. The metabolomics data collected for GnRH (Ctrl, PBS + GnRH, GnRH + GnRH) and GnIH (Ctrl, PBS + GnIH, GnIH + GnIH) treatments were analyzed independently to reduce the impact of artifacts and maximize the predictive ability of multivariate analysis. A standard library of retention times and *m/z*, with Maven software, led to the identification of 64 metabolites in the GnRH treated and 59 metabolites in the GnIH treated groups. PLS-DA was performed on the three treatment groups (control, single and double hormone injection) for each reproductive phase to investigate differences in the metabolic profiles of the three groups tested (Figure 6). PLS-DA models built for GnRH treatments show a statistically significant difference in the metabolic profile of the three treatment groups in the regressed (*p*-value = 0.001) and mid recrudescence (*p*-value = 0.046; Table 3) stages. On the other hand, GnRH treatment in the late recrudesce and GnIH treatment in all reproductive stages did not result in a statistically significant PLS-DA model for various treatment groups (Table 3). Variable selection based on the VIP score >1 criterion identified 26 metabolites in the regressed stage, 25 in the mid and 24 in the late recrudescence for the GnRH treatment groups (Figure 7A). For the GnIH treated groups, variable selection based on the VIP score >1 criterion identified 23 metabolites in the regressed stage, and 16 and 18 for the mid and late recrudesce, respectively (Figure 7B). The stacked bar graphs presented in Figure 7 show differences between models in terms of metabolite importance (VIP score >1), indicating different metabolic actions of hormones at different stages of gonadal recrudescence and providing a basis for comparing GnRH and GnIH effects. We employed multivariate and univariate statistical and visualization methods for each model’s VIP > 1 metabolites to investigate differences between treatment groups. To analyze the acute effect of GnRH and GnIH on seasonally-dependent basal metabolism, one clustered heatmap was built for each PLS-DA model highlighting the impact of single and double injection treatment (six in total, Figure 8). One-way ANOVA accompanied by multiple t-tests identified significant changes between experimental groups (Supplementary Tables S6-S13). Pathway analysis performed on the Metaboanalyst 4.0 platform identified metabolic pathways and physiological processes altered by treatments with GnRH and GnIH (Table 4). Within each reproductive phase, single and double hormone injected groups were compared independently to their respective control groups as the pathway analysis tool only allows the comparison between two groups at a time. The metabolic pathways impacted by single and double injection with GnRH or GnIH in the three stages of gonadal recrudescence are summarized in Table 4. Supplementary Tables S14, S15 provides more details on pathways analysis results.

**TABLE 3:** Statistical values for the Partial Least Squares—Discriminant Analysis (PLS-DA) models investigating the effect of GnRH and GnIH in the three investigated reproductive stages (Regressed, Mid and Late). Each model represents the comparison of three groups: control, single and double injection with **GnRH** or **GnIH**. *R* ^2^ and *Q* ^2^ parameters measure the “goodness of fit” and “goodness of prediction” respectively for each model. Statistical significance (*p*-value < 0.05) of models was assessed *via* Cross Validated—ANOVA (CV-ANOVA) based on a 7-fold cross validation permutation testing method.

GnRH			
Model	*R* ^2^	*Q* ^2^	*p*-value
Regressed	0.39	0.296	0.001
Mid	0.393	0.279	0.046
Late	0.395	0.117	0.097
**GnIH**			
Regressed	0.618	0.195	0.579
Mid	0.306	0	0.99
Late	0.626	0.282	0.99

**TABLE 4:** Summary of the main altered metabolic pathways resulted from pathway analysis investigating the effect of single and double injection with GnRH (above) and GnIH (below) during the three investigated reproductive stages (Regressed, Mid and Late).

GnRH								
Regressed			Mid			Late		
Metabolic pathway	PBS + GnRH	GnRH + GnRH	Metabolic pathway	PBS + GnRH	GnRH + GnRH	Metabolic pathway	PBS + GnRH	GnRH + GnRH
Purine metabolism	2.71 ×10^−7^	0.1783	Glycerophospholipid metabolism	0.0005	0.4777	Citrate cycle (TCA cycle)	0.0006	0.3684
Ketone bodies metabolism	0.0175	0.1322	Glycerolipid metabolism	0.0011	0.4256	Nucleotide sugar metabolism	0.0258	0.2765
Butanoate metabolism	0.0175	0.1322	Histidine metabolism	0.0038	0.3139	Fructose and mannose metabolism	0.0274	0.1099
Pyrimidine metabolism	0.0257	0.1479	beta-Alanine metabolism	0.0038	0.3139	Glycolysis/Gluconeogenesis	0.0274	0.1099
Nucleotide sugar metabolism	0.0347	0.8826	Nucleotide sugar metabolism	0.0100	0.7549	Ala, Asp and Glu metabolism	0.0332	0.0354
Starch and sucrose metabolism	0.0548	0.3393	Aminoacyl-tRNA biosynthesis	0.0247	0.5035	Arginine biosynthesis	0.0699	0.0461
Cys and Met metabolism	0.0591	0.7977	Ala, Asp and Glu metabolism	0.0269	0.7510	Ketone bodies metabolism	0.3557	0.0519
Gly, Ser and Thr metabolism	0.0591	0.7977	Pyrimidine metabolism	0.0282	0.8588	Val, Leu and Ile degradation	0.3557	0.0519
Galactose metabolism	0.0653	0.3897	Arginine biosynthesis	0.0382	0.7014	Tyrosine metabolism	0.3557	0.0519
Ala, Asp and Glu metabolism	0.2830	0.1446	Purine metabolism	0.0422	0.3811	Butanoate metabolism	0.1174	0.0520
Arginine biosynthesis	0.2830	0.1446	Pentose phosphate pathway	0.1051	0.0397	Purine metabolism	0.0785	0.0639
Gln and Glu metabolism	0.2830	0.1446	Tau and hypotaurine metabolism	0.1557	0.0297	Starch and sucrose metabolism	0.1230	0.1476
Pentose phosphate pathway	0.2939	0.1893	Ketone bodies metabolism	0.3316	0.0166	Galactose metabolism	0.1179	0.1502
			Butanoate metabolism	0.3316	0.0166	Glycerophospholipid metabolism	0.0682	0.2940
			Starch and sucrose metabolism	0.5779	0.0405	Glycerolipid metabolism	0.0682	0.2940
			Galactose metabolism	0.5779	0.0405	Tau and hypotaurine metabolism	0.1665	0.3996
			Val, Leu and Ile degradation	0.6408	0.0142			
			Tyrosine metabolism	0.6408	0.0142			

GnIH								
Regressed			Mid			Late		
Metabolic pathway	PBS + GnIH	GnIH + GnIH	Metabolic pathway	PBS + GnIH	GnIH + GnRH	Metabolic pathway	PBS + GnIH	GnIH + GnIH
Aminoacyl-tRNA biosynthesis	0.0306	0.2793	Ala, Asp and Glu metabolism	0.1954	0.0124	Histidine metabolism	0.0287	0.4272
Ala, Asp and Glu metabolism	0.0310	0.1733	Aminoacyl-tRNA biosynthesis	0.2608	0.0121	beta-Alanine metabolism	0.1954	0.4272
Gly, Ser and Thr metabolism	0.0358	0.2188	Pyrimidine metabolism	0.3752	0.0230	Purine metabolism	0.0307	0.2047
Galactose metabolism	0.4527	0.0286	Gln and Glu metabolism	0.3617	0.0216	Pyrimidine metabolism	0.0046	0.0061
Purine metabolism	0.5252	0.0009	Arginine biosynthesis	0.3617	0.0216	Arginine biosynthesis	0.461	0.0356
Arg and Pro metabolism	0.0628	0.3981	Nitrogen metabolism	0.3617	0.0216	Propanoate metabolism	0.1462	0.0277
Phe, Tyr and Trp biosynthesis	0.0944	0.4458	Pantothenate and CoA biosynthesis	0.5322	0.0084	Synthesis of unsaturated fatty acids	0.1842	0.0186
Tyrosine metabolism	0.0944	0.4458	Purine metabolism	0.6246	0.0133	Butanoate metabolism	0.2504	0.0108
Arginine biosynthesis	0.1445	0.2148	Gly, Ser and Thr metabolism	0.0502	0.1918	Ketone bodies metabolism	0.2841	0.0279
Starch and sucrose metabolism	0.2016	0.0522	Arginine and proline metabolism	0.0695	0.2354	Citrate cycle (TCA cycle)	0.5722	0.0083
Pentose phosphate pathway	0.2138	0.9407	Starch and sucrose metabolism	0.5782	0.0852	Glycerophospholipid metabolism	0.7752	0.0101
Glutathione metabolism	0.2604	0.5881				Tyrosine metabolism	0.7836	0.0166
Citrate cycle (TCA cycle)	0.3507	0.2370				Pyruvate metabolism	0.7836	0.0166
						Gly, Ser and Thr metabolism	0.8491	0.0496
						Cys and Met metabolism	0.8491	0.0496
						Sphingolipid metabolism	0.9411	0.0078

Values indicate the *p*-value obtained from the independent comparison between control and single injection groups (PBS + GnRH or PBS + GnIH) and control and double injection groups (GnRH + GnRH or GnIH + GnIH). *p*-values in red indicate significantly altered metabolic pathways (*p*-value < 0.05). The detailed results of pathway analysis for each comparison are shown in Supplementary Tables S14, S15.

#### 3.2.1 Regressed Gonadal Phase—Growth Phase

During the regressed phase corresponding to the period of maximal growth, a single injection with GnRH significantly impacted purine and pyrimidine metabolism (Table 4). As shown in the heatmap (Figure 8) and confirmed by ANOVA results (Supplementary Table S6), hypoxanthine, xanthine, and uric acid were significantly increased following a single GnRH injection, compared to the control, suggesting elevated purine degradation. Interestingly, in the regressed phase, purine metabolism was also significantly altered by double injection with GnIH (Table 4). Single GnRH injection and single and double injections with GnIH increased purine degradation by inducing higher hypoxanthine, xanthine, and uric acid levels than the control (Figure 8). It should be noted that basal hypoxanthine, xanthine and uric acid levels were also higher in the late recrudescence than regressed and mid recrudescence, indicating that GnRH and GnIH treatment in the regressed phase can alter the pathways to resemble that observed during late recrudescence. As shown in the heatmap, a single injection with GnRH decreased the concentration of several carbohydrates such as glycogen, raffinose, oligomaltose, sucrose, fructose, glucose and glucose 6-phosphate (Figure 8). However, based on ANOVA, only differences for sucrose, fructose and glucose were statistically significant (Supplementary Table S6), and starch, sucrose and galactose metabolism were not significantly impacted (Table 4). Furthermore, single injection with GnRH enhanced ketone bodies metabolism resulting in a higher concentration of 3-hydroxybutyric acid than the control, as highlighted by the heatmap (Figure 8). Unlike single injection, double injection with GnRH did not change the metabolic pathways (Table 4), and the level of metabolites shown in the heatmap are similar to the control (Figure 8). Single injection with GnIH during the regressed phase also altered the metabolism of several amino acids, including alanine, aspartate, glutamate, glycine, serine and threonine metabolism, while double injection with GnIH significantly impacted the metabolism of carbohydrates, including galactose (Table 4). Accordingly, sucrose concentration was decreased by both single and double injection with GnIH (Figure 8). Single and double GnIH injection increased creatine level (Figure 8), suggesting the involvement of GnIH in the energy homeostasis. Creatine and its phosphorylated form function as an energy buffer facilitating ATP recycling to support the cellular energy balance (Borchel et al., 2019).

#### 3.2.2 Mid Recrudescence

Pathway analysis during mid recrudescence revealed different metabolic pathways affected following single and double injection with GnRH (Table 4). This is supported by the VIP > 1 metabolites’ heatmap, also showing a significantly different profile between single and double GnRH-injected groups (Figure 8). Single GnRH injection significantly altered metabolic pathways connected to the lipid metabolism (glycerophospholipid and glycerolipid metabolism; Table 4), which correlated with significantly lower levels of *O*-phosphoethanolamine (EAP) compared to the control (Figure 8; Supplementary Table S6). Single injection with GnRH also altered the purine and pyrimidine metabolism (Table 4). In this context, changes in the concentration of relevant metabolites indicate decreased purine and pyrimidine synthesis and increased purine degradation (Figure 8). Double injection with GnRH altered pathways connected to the carbohydrate metabolism. We observed significant changes in starch, sucrose, galactose, ketone bodies metabolism as well as the metabolism of several amino acids, including taurine, valine, leucine, isoleucine and tyrosine (Table 4). As for GnIH, single injection during mid recrudescence did not significantly change metabolic pathways annotated in the KEGG Danio rerio database (Table 4). However, double injection with GnIH significantly altered the metabolism of purine and pyrimidine, pantothenate, and the metabolism of several amino acids (Table 4). As shown in the heatmap, double injection with GnIH also increased the glucose and glucose 6-phosphate and decreased glutamine levels, compared to control (Figure 8; Supplementary Table S10).

#### 3.2.3 Late Recrudescence

Single injection with GnRH in late recrudescence altered pathways related to carbohydrate and energy metabolism such as fructose and mannose metabolism, glycolysis/gluconeogenesis and TCA cycle (Table 4). On the other hand, double injection with GnRH impacted the metabolism of amino acids such as alanine, aspartate, glutamate, and arginine (Table 4). Also shown in the heatmap of VIP > 1 metabolites, single and double injection with GnRH decreased the levels of carbohydrates such as glycogen, raffinose, oligomaltose and sucrose, while increasing the levels of glucose, fructose, dihydroxyacetone phosphate (DHAP) and succinic acid (Figure 8). These results suggest that GnRH promotes the degradation of carbohydrate storage to be used as an energy source *via* glycolysis and the TCA cycle. In the case of GnIH, both single and double injections affected similar pathways, although double injection exerted greater effects than single injection (Figure 8). As shown in the heatmap of VIP > 1 metabolites, the concentrations of several metabolites, including raffinose, fumaric acid and succinic acid, were progressively reduced following single and double treatment with GnIH (Figure 8). In this context, pathways related to energy metabolism (propanoate and pyruvate metabolism, TCA cycle) were impacted to a greater extent by double injection than single injection with GnIH (Table 4). Double injection with GnIH significantly affected lipid metabolism by altering pathways such as biosynthesis of unsaturated fatty acids, glycerolipid and sphingolipid metabolism (Table 4). Changes in the concentration of relevant metabolites such as docosahexaenoic acid (DHA) and *O*-phosphoethanolamine (EAP) suggest a stimulatory action of GnIH on these metabolic pathways (Figure 8). Furthermore, double injection with GnIH significantly inhibited ketone bodies metabolism as the levels of 3-hydroxybutiric acid are decreased by single and double injections (Figures 8).

## 4 Discussion

In the present study, we used an LC-MS-based targeted metabolomics approach to investigate the hepatic metabolic profile of sexually mature female goldfish in three stages of the reproductive cycle, including the regressed gonadal stage corresponding to the period of maximal growth as well as mid and late gonadal recrudescence. Moreover, we investigated the effects of acute injections with GnRH and GnIH on liver metabolism to explore the potential involvement of these hormones in the control of energy allocation during growth and reproduction in goldfish. The liver was chosen as a tissue of interest since it is the primary energy hub and is responsive to hormonal and metabolic signals, influencing the whole-body metabolism (Rui, 2014). Furthermore, the liver is the site of Vtg synthesis, which is a precursor molecule for yolk proteins (Reading and Sullivan, 2011).

### 4.1 Metabolic Changes During Growth and Reproductive Phase

The multivariate and univariate analysis allowed the identification of significant changes in the hepatic metabolic profile of female goldfish during different stages of the reproductive cycle. Based on the observed changes in the levels of metabolites and active metabolic pathways, we were able to identify changes in amino acid, carbohydrate, lipid, nucleotide, and energy metabolism during growth and reproductive cycles.

#### 4.1.1 Regressed Gonadal Phase is Characterized by Growth-Promoting Processes

Maximal growth is observed during the regressed gonadal phase in fish, and our results demonstrate an enhanced nucleotide and protein metabolism during this period. Higher concentrations of amino acids including tyrosine, arginine, alanine, lysine, leucine, valine, methionine, histidine, tryptophan and phenylalanine indicate an increased amino acids metabolism and turnover possibly in support of enhanced protein synthesis (Figure 4; Table 2). The observed higher concentration of tyrosine in regressed, compared to mid and late recrudescence, is correlated with higher circulating thyroid hormones (T3 and T4) in the regressed goldfish (Sohn et al., 1999). Our results combined with observed higher thyroid hormone receptor (TRβ) mRNA level in the regressed female goldfish liver (Ma et al., 2020b), provide further support for the hypothesis that thyroid hormones are essential components of growth response during the regressed gonadal phase in fish. It should be noted that the role of thyroid hormones in the control of growth is widely recognized (Cabello and Wrutniak, 1989; Nelson and Habibi, 2009). Thyroid hormones regulate GH and IGF-1 production as well as liver GH receptor synthesis in several vertebrates (Tsukada et al., 1998; Schmid et al., 2003). Besides interacting with the somatotropic axis, thyroid hormones can directly stimulate growth by enhancing protein synthesis and regulating glucose homeostasis. T3 was shown to increase protein synthesis at a pre-translational level through stimulation of ribosomal and nuclear RNA synthesis (Müller and Seitz, 1984). We hypothesize that the enhanced amino acid metabolism demonstrated during the regressed phase results from increased protein synthesis stimulated by thyroid hormones and GH. These hormones are present at higher levels during the regressed phase (Marchant and Peter, 1986; Sohn et al., 1999). Thyroid hormones stimulate enzymes of the gluconeogenic pathway, resulting in increased plasma glucose levels in several fish species (Deal and Volkoff, 2020). The hyperglycemic effect of thyroid hormones may be a contributing reason for higher concentrations of glucose and fructose observed in the present study during the regressed phase, compared to late recrudescence (Figure 4; Table 2). Increased glucose utilization would support protein synthesis by enhancing energy metabolism and synthesis of amino acids from intermediates of the TCA cycle. This is validated by the pathway analysis results comparing regressed and mid-recrudescent fish that show a significant impact on pathways related to energy metabolism, such as pyruvate metabolism and the TCA cycle (Figure 5). We hypothesize that the observed increases in taurine and hypotaurine metabolism in the growth stage result from increased anabolic pathways activity (Figure 5). The sulphur-containing amino acid taurine plays a crucial role in supporting growth-related processes by modulating metabolism and nutrient utilization (Salze and Davis, 2015; de Moura et al., 2018). Taurine effects on growth and reproductive performances have been widely explored in fish nutrition. In several teleost species, dietary taurine supplementation improved glucose metabolism by increasing gene expressions and activity of enzymes involved in glycogen synthesis and anabolic pathways (Sampath et al., 2020). Thus, taurine may contribute to the enhanced glycogen synthesis observed during the regressed phase (Figure 4; Table 2).

#### 4.1.2 Metabolic Profile During Mid and Late Recrudescence

The investigation of the metabolic profile of mid and late recrudescence highlighted differences concerning amino acid, nucleotide, lipid and carbohydrate metabolism. In accordance with previous studies conducted on goldfish (Delahunty et al., 1978), common roach, white bream (Rinchard and Kestemont, 2003), singi fish (Dasmahapatra and Medda, 1982) and flounder (Petersen and Emmersen, 1977), our results demonstrate a significantly lower concentration of glycogen in mid recrudescence compared to regressed phase. This result suggests an elevated breakdown of glycogen as an energy source to support the vitellogenic process. This is consistent with the observed higher glucose 1-phosphate in mid compared to late recrudescence (Figure 4; Table 2). Furthermore, the elevated degradation of glycogen can be correlated to the higher circulating estrogen and liver estrogen receptor levels measured in mid recrudescence compared to other reproductive stages (Nelson and Habibi, 2013; Ma et al., 2020b). Accordingly, injections of estrogen in singi fish reduced glycogen and increased liver lipid content of vitellogenic female fish (Dasmahapatra and Medda, 1982). Our results also demonstrate enhanced lipid metabolism, particularly glycerophospholipid and sphingolipid metabolism and fatty acids biosynthesis, in mid compared to late recrudescence (Figure 5). This is consistent with elevated levels of *O*-phosphoethanolamine and malonate in mid compared to late recrudescence (Table 2). Lipovitellin, a major product of Vtg degradation, is the main component in fish egg yolk and has a lipid content of about 20% by mass, primarily consisting of phospholipids and triglycerides (Hara et al., 2016). The lipid content of ovaries from wild Tilapia nilotica ranged from 12.2% to 25.5% of the wet weight (James Henderson and Tocher, 1987). We hypothesize that the enhanced lipid metabolism in the liver of mid-recrudescent fish characterizes the peak vitellogenic period, during which the mobilization of lipid from the liver is essential to support gonadal maturation. Accordingly, transcript levels of vitellogenin in goldfish liver in significantly higher in mid compared to regressed fish (Ma et al., 2020b). Furthermore, the serum levels of total lipids and phospholipids were elevated during flounder vitellogenesis (Petersen and Emmersen, 1977). Significantly higher ornithine and citrulline levels were also observed in mid compared to late recrudescence (Figure 4; Table 2). These metabolites derive from arginine and proline catabolism and are precursors in the synthesis of polyamines such as spermidine and spermine, known to be essential for the success of several reproductive functions in mammals (Lefèvre et al., 2011). Polyamine synthesis seems necessary for the function and differentiation of the somatic cell component of the ovary (Lefèvre et al., 2011). Furthermore, studies on *Xenopus* oocytes indicate that polyamines are essential for cytoplasmic maturation and protect the oocyte in the metaphase II stage from ROS-induced apoptosis (Bassez et al., 1990). In line with this, our results demonstrate enhanced polyamine synthesis during mid recrudescence, indicating the importance of these molecules in the reproductive cycle of female goldfish. The heatmap and multiple t-test results indicate significantly lower concentrations of several essential amino acids, including histidine, tryptophan, arginine, lysine, leucine, valine, methionine, phenylalanine and threonine in late recrudescence compared to the regressed phase (Figure 4; Table 2). Studies investigating the composition of marine pelagic fish eggs showed an abrupt increase in free amino acids content shortly before ovulation, reaching a total amino acid content of 40–60% of dry mass in newly spawned eggs (Rønnestad et al., 1999). In another study by Qari et al. (2013) higher concentrations of essential amino acids were observed in the ovaries of ripe goldlined seabream compared to nearly ripe fish. The lower liver content of amino acids in late compared to the regressed phase described in this study suggests protein mobilization from the liver to the ovaries as the fish reaches maturation and highlights the importance of essential amino acids in the reproductive phase of the annual cycle. Pathway analysis results indicate that late recrudescence is also characterized by significantly altered purine metabolism compared to mid recrudescence (Figure 5). Accordingly, the heatmap shows higher levels of hypoxanthine, xanthine, uric acid and allantoin (Figures 4, 5) that are metabolites of the degradation pathway of purine metabolism. In fish, purine degradation ends with the oxidation of uric acid to allantoin by the enzyme urate oxidase (Kinsella et al., 1985). Uric acid is an important antioxidant in humans, and several studies have demonstrated its potent ability as a scavenger of oxygen singlet and hydroxyl radicals (Kand’ár et al., 2006). In the liver of mildly diabetic rats, uric acid and xanthine concentration were threefold and sixfold higher than control, respectively, revealing the role of these molecules in the homeostatic response to the oxidative stress (Kristal et al., 1999). Studies on the inclusion of nucleotides in fish diet have demonstrated they may increase oxidative stress tolerance in part by offsetting the inhibitory effect of cortisol on the fish immune system (Li and Gatlin, 2006). Furthermore, red sea bream fed diets with different content of guanosine monophosphate (GMP) resulted in a lower intensity of oxidative stress and higher antioxidant capacity against the oxidative stress (Hossain et al., 2016). Glutathione was also shown to have significantly higher concentration levels in late than regressed and mid recrudescence (Figure 4; Table 2). Accordingly, glutathione metabolism was found to be significantly different in mid/late and late/regressed pathway analysis results (Figure 5). Glutathione is the most abundant and important intracellular antioxidant, produced mainly in the liver from glutamate, glycine and cysteine (Wu, 2009). Reproduction is an energy-demanding process resulting in changes in the balance between pro-oxidant and antioxidant molecules due to increased metabolic rates (Alonso-Alvarez et al., 2004). Gonadotropins, testosterone and estrogen directly interact with energy metabolism and influence oxidative balance and glutathione metabolism (Lamartiniere, 1981; Norris and Hobbs, 2011). Indeed, total antioxidant capacity and antioxidant enzymes activity in the liver of sea trout were higher in the adult and spawner stages than post-spawning fish (Kurhaluk, 2019). Although elevated oxidative stress can impair reproduction, reactive oxygen species (ROS) are important signalling molecules in oocyte maturation. *In vivo* and *ex vivo* studies on mice ovaries show that ROS present in the preovulatory ovarian follicles are essential for ovulation and are most likely caused by the preovulatory LH surge induced inflammatory response (Shkolnik et al., 2011). Similarly, follicular ROS generated by NADPH oxidase is essential for ovulation in *Drosophila* and suggests a conserved role in regulating ovulation in other species (Li et al., 2018). Based on our results, we hypothesize that elevated liver content of antioxidant molecules such as uric acid and glutathione in late recrudescence compared to mid and regressed female fish is necessary to protect the liver from the potential oxidative damage induced by the preovulatory surge of LH. In contrast to what was measured in the liver of male goldfish (Ladisa et al., 2021), the concentration of acetoacetic acid is significantly lower in late compared to regressed and mid recrudescence (Table 2). This is accompanied by lower levels of 3-hydroxybutyrate in mid and late recrudescence, compared to regressed (Figure 4). Accordingly, ketone bodies metabolism and butanoate metabolism were found to be different in our pathway analysis comparisons (Figure 5). Our results indicate that the synthesis of ketone bodies is downregulated in mid and late recrudescence compared to the regressed stage. Little information is available regarding the involvement of ketone bodies metabolism in fish growth and reproduction. In mammals, high ketone bodies are typical during starvation since they function as energy sources derived from accelerated fatty acid degradation (Fukao et al., 2004). Previous studies on mammals have demonstrated the role of ketone bodies in regulating food intake and energy homeostasis by showing that central infusion of β-hydroxybutyrate increased food intake in rats (Iwata et al., 2011b) and mice (Carneiro et al., 2016). However, starvation of carp (Segner et al., 1997) bass (Zammit and Newsholme, 1979), and rainbow trout (Jonas and Bilinski, 1965) did not affect food intake or ketogenesis. Together these results suggest that in fish ketone bodies metabolism might be less important in the regulation of food intake when compared to mammals and might have a significant role in other physiological processes. Injection of β-hydroxybutyrate in the brain of female rats suppressed the pulsatile secretion of LH (Iwata et al., 2011a). Finally, higher than normal serum and milk levels of β-hydroxybutyrate during lactation in dairy cows results in a delayed start of the postpartum ovarian cycle (Koller et al., 2003). Based on our results, we hypothesize that low levels of ketone bodies as a consequence of reduced liver ketogenesis in late recrudesce compared to the regressed phase is a permissive/stimulating mechanism for the pre-spawning surge of LH. This hypothesis can be linked to the significantly higher levels of circulating LH recorded in female goldfish during late recrudescence than regressed and mid recrudescence (Ma et al., 2020b).

### 4.2 Acute Effect of Gonadotropin-Inhibitory Hormone and Gonadotropin-Releasing Hormone on Liver Metabolism of Regressed, Mid and Late Recrudescence Fish

Metabolomics results presented in this study indicate that treatments with GnRH induced significant changes during regressed and mid recrudescence but not during late recrudescence, as measured by the PLS-DA regression models (Table 3). On the other hand, PLS-DA models for GnIH treatments did not indicate significant changes between groups in any investigated reproductive stages (Table 3). In this study, we did not investigate the time-related effects of GnRH and GnIH on metabolism, and the acute nature of the treatments may have affected metabolic pathways to a lesser extent than that observed at different periods of the reproductive cycle. It is possible that the PLS-DA models constructed on control, single and double injected groups may not have identified all the changes that would be induced by more prolonged exposure to GnRH and GnIH treatments. However, variable selection based on the criteria of VIP score >1 is still a robust method for identifying variables that influence the separation between groups and characterize changes in the metabolic profile (Gromski et al., 2014).

#### 4.2.1 Regressed Gonadal Phase Corresponding to Maximal Growth Phase

During the regressed gonadal stage, single and double injections with GnRH had different effects on liver metabolism. Pathway analysis identified significant changes in metabolic pathways after a single injection with GnRH (Table 4). However, double injection with GnRH did not affect pathways noted in the zebrafish KEGG database (Table 4). Accordingly, the metabolic profile following double injection in the heatmap is similar to the control (Figure 8). A similar study in goldfish demonstrated that single but not double injection with GnRH significantly increased circulating GH levels and decreased Erα, ERβ1 TRα and TRβ mRNA liver transcript levels in the regressed female goldfish (Ma et al., 2020b). In the present study, single injection with GnRH significantly altered purine and pyrimidine metabolism due to decreased levels of guanosine, adenosine, uridine, cytidine and inosine and increased levels of hypoxanthine, xanthine and uric acids (Figure 8; Table 4). Moreover, the clustered heatmap shows lower levels of several carbohydrates such as glycogen, raffinose, sucrose, oligomaltose, fructose, glucose and glucose 6-phosphate in the single GnRH injected group, compared to the control (Figure 8). Similar changes in purine and carbohydrates metabolism were observed in fish at late recrudesce, compared to regressed and mid recrudescence (Figure 4). Single and double GnIH injection treatments affected different pathways than the control (Table 4). According to the heatmap results, single and double injection with GnIH progressively increased the concentrations of several metabolites, including hypoxanthine, xanthine, uric acid and sucrose (Figure 8). This is consistent with a previous study that demonstrated that single and double GnIH injection significantly altered the liver ERα, ERβ1, TRβ and IGF-I transcript levels (Ma et al., 2020b). Double injection with GnIH at the regressed phase significantly inhibited purine and carbohydrate metabolism (Table 4), resulting in increased hypoxanthine and xanthine levels and decreased concentration of sucrose and glucose 6-phosphate (Figure 8). Together, our results indicate that GnIH affects reproduction, in part, by influencing metabolic pathways related to the late recrudescence stage.

#### 4.2.2 Mid Recrudescence

Pathway analysis revealed significant differences between single and double injection effects with GnRH. Single injection with GnRH inhibited amino acid and lipid metabolism pathways and enhanced purine degradation and ketone bodies metabolism (Figure 8; Table 4). However, double injection with GnRH stimulated ketone bodies synthesis and increased the concentrations of several amino acids, including asparagine, aspartic acid and serine, and inhibited carbohydrates metabolism (Figure 8; Table 4). In general, single GnRH injection inhibited metabolic pathways related to reproductive processes, whereas double injection enhanced the pathways related to reproduction at mid recrudescence (Table 4). This is consistent with the observation that single GnRH injection decreased Vtg and IGF-I mRNA levels, which was increased following double injection with GnRH in goldfish at mid recrudescence (Ma et al., 2020b). In the present study, double injection with GnRH also enhanced taurine and hypotaurine metabolism (Figure 8; Table 4). Previous studies identified taurine as an essential component of broodstock diets for Japanese yellowtail (Matsunari et al., 2006) and greater amberjack (Sarih et al., 2019), necessary to improve fecundity, egg viability and fertilization rates. Thus, our results demonstrate a potential role of GnRH in stimulating metabolic pathways favouring reproductive processes. Furthermore, double injection with GnIH also altered metabolic pathways related to amino acid, pyrimidine and purine metabolism (Table 4). The clustered heatmap suggests that the effect of GnIH is dose-dependent and double injection with GnIH potentiates the effect induced by single injection treatment (Figure 8). Similar results were obtained after treatments with GnIH during the regressed phase (Figure 8). The metabolic changes induced by GnIH treatments characterize the basal metabolic profile of regressed fish, compared to mid and late recrudescence (Figures 4, 8). The results suggest that treatments with GnIH in mid recrudescence inhibit reproductive processes and stimulate metabolic pathways related to the growth phase.

#### 4.2.3 Late Recrudescence

The pathway analysis demonstrates that GnRH injections alter carbohydrate and energy metabolism in late recrudescence (Table 4). Single and double injection with GnRH resulted in lower levels of carbohydrates such as glycogen, raffinose, oligomaltose and sucrose and higher levels of glucose, fructose, dihydroxyacetone phosphate (DHAP) and succinic acid. Injection with GnRH stimulated carbohydrate mobilization and energy production during late recrudescence (Figure 8). Observed changes in late recrudescence indicate that GnRH stimulates metabolic processes related to vitellogenesis and gonadal maturation. Our results are consistent with reported stimulation of circulating LH concentration and increased expression of ERα and ERβ1 mRNA following injection with GnRH in female goldfish at late recrudescence (Ma et al., 2020b). Similarly, GnIH stimulated metabolic pathways related to the mid recrudescence stage. The clustered heatmap demonstrates a progressive decrease in glycogen, raffinose, fumaric acid, succinic acid, and 3-hydroxybutyrate stimulated following GnIH treatments. Single and double injection with GnIH also reduced the levels of *O*-phosphoethanolamine (EAP) and docosahexaenoic acid (DHA; Figure 8; Supplementary Table S10).

## 5 Conclusion

Results from this study characterize the liver metabolic profile of female goldfish at three stages of the reproductive cycle. In the regressed gonadal phase, carbohydrate and amino acid metabolism were enhanced, possibly supporting anabolic processes related to growth. On the other hand, the liver metabolism of mid-recrudescent fish is characterized by elevated lipid synthesis during the peak of vitellogenesis and lipid mobilization from the liver to support maximal gonadal maturation. The metabolic profile of goldfish during late recrudescence revealed a high concentration of antioxidant molecules such as uric acid and glutathione and reduced ketone bodies. These molecules may play a vital role in the final stages of the gonadal cycle. In the present study, we also demonstrated that GnRH and GnIH are involved in the multifactorial regulation of metabolism in supporting growth and reproduction in female goldfish. Results indicate that acute treatment with GnRH and GnIH induce metabolic changes depending on the season and mode of administration. GnRH treatments had similar effects during the regressed phase and mid recrudescence. Double injection with GnRH enhanced pathways related to growth and reproduction in regressed and mid recrudescence, respectively. On the other hand, GnRH treatments during late recrudescence stimulated pathways supporting vitellogenesis and gonadal maturation. GnIH showed an additive effect of single and double injections and changed the metabolic profile resembling the previous reproductive stage. During the regressed phase, GnIH treatments stimulated metabolic pathways related to the final stages of reproduction. In contrast, GnIH promoted a shift toward growth processes during mid recrudescence. Finally, during late recrudescence, GnIH stimulated metabolic pathways characteristic of the mid recrudescence metabolic profile. Overall, our results provide new information on the metabolic changes that accompany the growth and reproductive processes during the annual reproductive cycle of female goldfish and provide an insight into the role of GnRH and GnIH in the multifactorial control of growth and reproduction in goldfish and other oviparous species.

## Data Availability

The original contributions presented in the study are included in the article/Supplementary Material, further inquiries can be directed to the corresponding author.
