# Supplementary material for: Metabolic Changes During Growth and Reproductive Phases in the Liver of Female Goldfish (Carassius auratus)
Source: Front Cell Dev Biol. 2022 Feb 28;10:834688. doi: 10.3389/fcell.2022.834688 (PMC8919208; doi:10.3389/fcell.2022.834688)
Supplement: Supplementary file 1 [file DataSheet1.docx]

**Supplementary material**

**Metabolic changes during growth and reproductive phases in the liver of female goldfish (*Carassius auratus*)**

Claudia Ladisa, Yifei Ma and Hamid R Habibi

Department of Biological Sciences University of Calgary, 2500 University Drive NW, Calgary, Alberta, Canada T2N 1N4

**List of figures and tables**

**Figure S1**. Peak detection and quantification using MAVEN freeware.

**Figure S2**. Principal Component Analysis (PCA) score scatter plot.

**Table S1.** Mean intensity (± SEM) of the VIP>1 metabolites during the three investigates reproductive stages

**Table S2.** Pathway analysis (MetPa) results conducted on the concentration levels of the VIP>1 metabolites identified for the Regressed/ Mid PLS-DA model.

**Table S3.** Pathway analysis (MetPa) results conducted on the concentration levels of the VIP>1 metabolites identified for the Mid/ Late PLS-DA model.

**Table S4.** Pathway analysis (MetPa) results conducted on the concentration levels of the VIP>1 metabolites identified for the Late/ Regressed PLS-Da model.

**Table S5.** Multiple t-test results conducted on the VIP>1 metabolites identified for each PLS-DA model.

**Table S6.** One-way ANOVA results of the VIP>1 metabolites identified in the three PLS-DA models investigating the effect of GnRH in the three reproductive phases.

**Table S7.** Multiple t-test results conducted on the VIP>1 metabolites identified in the PLS-DA model investigating the effect of GnRH during the regressed gonadal phase

**Table S8.** Multiple t-test results conducted on the VIP>1 metabolites identified in the PLS-DA model investigating the effect of GnRH during mid recrudescence

**Table S9.** Multiple t-test results conducted on the VIP>1 metabolites identified in the PLS-DA model investigating the effect of GnRH during late recrudescence

**Table S10.** One-way ANOVA results of the VIP>1 metabolites identified in the three PLS-DA models investigating the effect of GnIH in the three reproductive phases.

**Table S11.** Multiple t-test results conducted on the VIP>1 metabolites identified in the PLS-DA model investigating the effect of GnIH during the regressed gonadal phase

**Table S12.** Multiple t-test results conducted on the VIP>1 metabolites identified in the PLS-DA model investigating the effect of GnIH during mid recrudescence

**Table S13.** Multiple t-test results conducted on the VIP>1 metabolites identified in the PLS-DA model investigating the effect of GnIH during late recrudescence

**Table S14.** Pathway analysis (MetPa) results investigating the effect of GnRH in the three reproductive phases.

**Table S15.** Pathway analysis (MetPa) results investigating the effect of GnIH in the three reproductive phases.


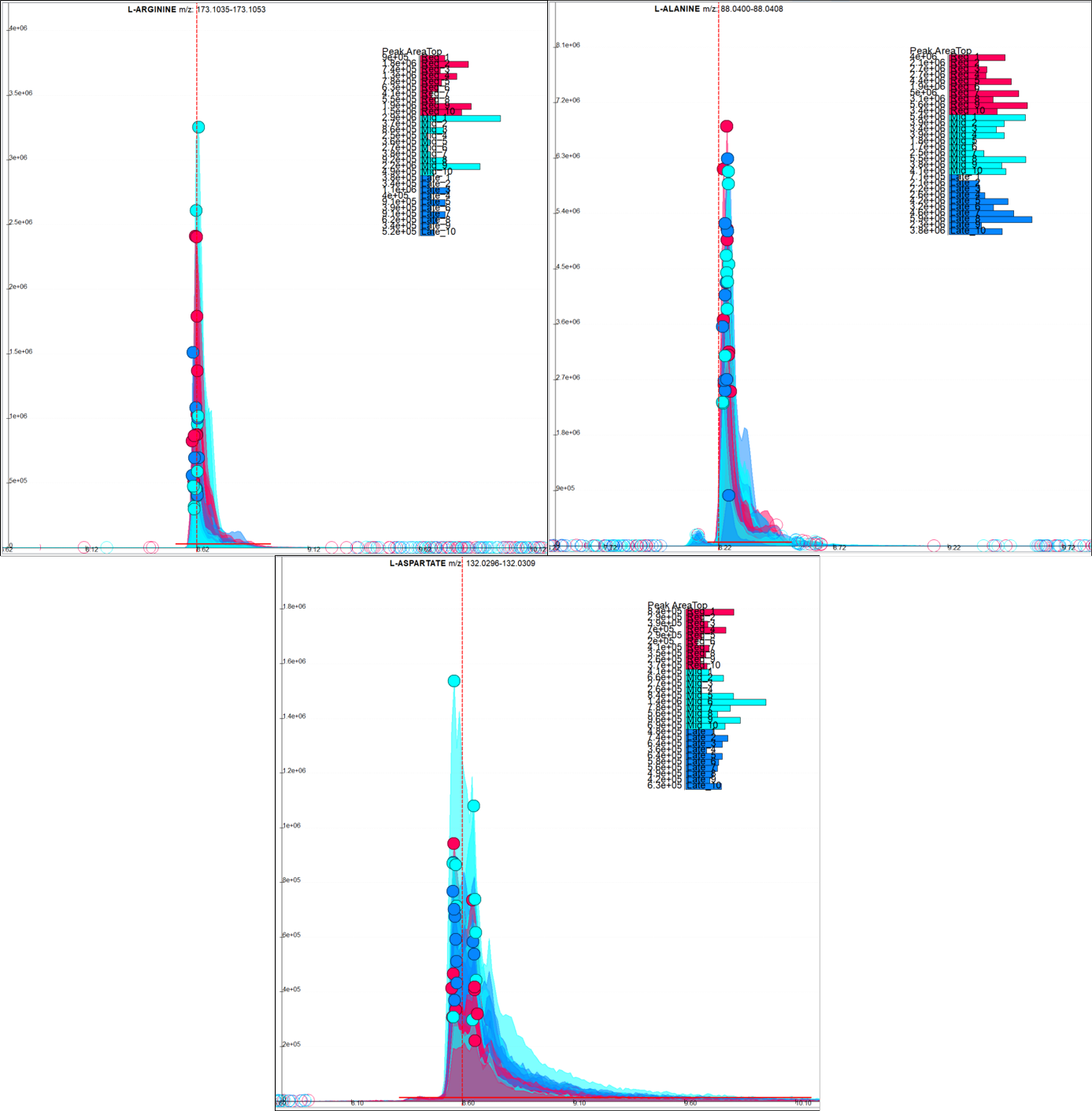


**Fig. S1.** Peak detection and quantification using MAVEN freeware. Examples of detected metabolites include Arginine, Alanine and Aspartate. The x-axis shows the retention time whereas the y-axis measures the peak intensity. The bar graph on the right of each peak represents the peak intensity metric *Area top.*

**
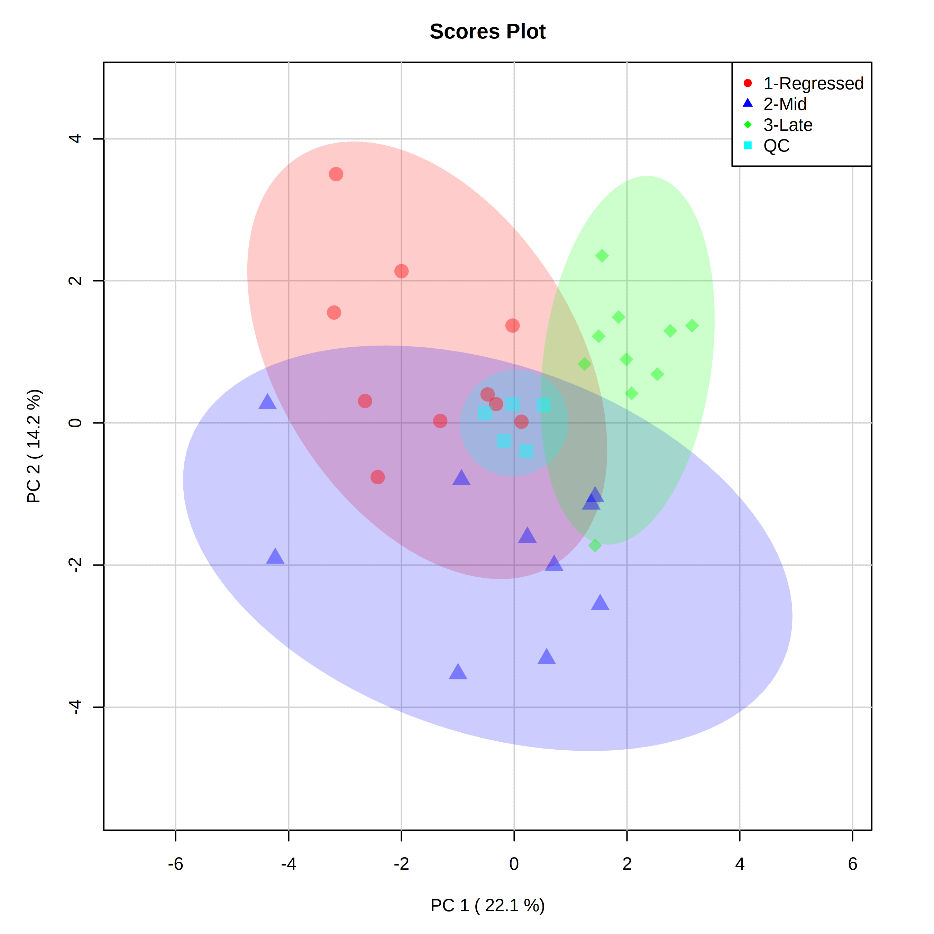
Fig. S2.** Principal Component Analysis (PCA) score scatter plot. PCA was performed on Regressed (regressed gonadal phase/somatotropic phase; n=10), Mid (mid gonadal recrudescence, n=10), Late (late recrudescence, n=10) and the Quality control group (n=5). Each axis represents a principal component (PC) that is a source of variation between samples. The percentage of total variance for each PC are shown in parentheses. The graph shows the absence of outliers as all samples are comprised in the 95% confidence interval of their respective group as well as a strong cluster formation for the QC group.

**Table S1.** Mean intensity (± SEM) of the VIP>1 metabolites during the three investigates reproductive stages.

|  | **Regressed** | | **Mid** | | **Late** | |
| --- | --- | --- | --- | --- | --- | --- |
| Metabolic pathway | Mean intensity | SEM | Mean intensity | SEM | Mean intensity | SEM |
| 3-Hydroxybutyric acid | 325300 | 45945.63 | 224400 | 16650.79 | 316400 | 34290.01 |
| Acetoacetic acid | 140370 | 27679.32 | 70340 | 17414.25 | 30717 | 12018.82 |
| Acetylglutamine | 43560 | 4794.724 | 94460 | 14831.61 | 45630 | 4334.104 |
| Adenine | 2352000 | 555151 | 613700 | 157890.3 | 1571300 | 179735.4 |
| Adenosine | 136070 | 39301.59 | 45020 | 7623.222 | 86890 | 10493.15 |
| Adipic acid | 685300 | 95976.39 | 1099400 | 134608.7 | 1200600 | 134645.9 |
| Alanine | 3495000 | 388896.6 | 3603000 | 414452.9 | 3156700 | 473311 |
| Allantoin | 607100 | 186590.8 | 343120 | 65204.13 | 1014000 | 170106.2 |
| Aminoadipic acid | 42140 | 5530.967 | 97080 | 22885.37 | 68550 | 13203.87 |
| AMP | 842600 | 147832.4 | 1161700 | 120589.7 | 765000 | 72886.52 |
| Arginine | 1047200 | 165553.8 | 898800 | 288937.5 | 588000 | 86357.14 |
| Asparagine | 161600 | 15136.56 | 318500 | 19476.62 | 275100 | 27445.89 |
| Aspartic acid | 410500 | 64219.46 | 681900 | 108277.8 | 553600 | 36539.84 |
| Azelaic acid | 102640 | 33704.67 | 98680 | 42571 | 56120 | 5605.648 |
| Ciliatine | 64457 | 11877.95 | 76479 | 16273.69 | 27974 | 6725.653 |
| Citrulline | 180590 | 45610.59 | 409320 | 91258.35 | 142140 | 48913.49 |
| CMP | 31814 | 7908.992 | 55750 | 7476.649 | 17006 | 5338.083 |
| Creatine | 2934000 | 326446.2 | 3067000 | 344661 | 2629800 | 399488.6 |
| EAP | 404900 | 38894.86 | 481200 | 36323.48 | 445600 | 47340.66 |
| Fructose | 27390000 | 2880411 | 24130000 | 1536667 | 27550000 | 1115771 |
| Fumaric acid | 5994000 | 647038 | 4099000 | 343849 | 6696000 | 472212.8 |
| Glucose | 34940000 | 3352452 | 30640000 | 1928338 | 33800000 | 1086176 |
| Glucose 1-phosphate | 2325600 | 261386.5 | 2901000 | 254167.3 | 2091900 | 225720.7 |
| Glutamic acid | 32980000 | 2567307 | 38230000 | 1567450 | 30800000 | 2611513 |
| Glutathione | 72320 | 32590.07 | 277600 | 131725.4 | 913270 | 193205.9 |
| Glycogen | 223430 | 39262.48 | 296800 | 33720.02 | 260900 | 23900.46 |
| GMP | 252400 | 12860.1 | 346000 | 23605.55 | 300100 | 19357.71 |
| Guanosine | 942700 | 153445.7 | 526700 | 67079.4 | 813700 | 106756.9 |
| Histidine | 1030200 | 63661.92 | 986300 | 82188.41 | 1014900 | 66703.4 |
| Homoserine | 837700 | 165664.7 | 664000 | 162817.4 | 702800 | 130316.9 |
| Hypotaurine | 142360 | 31041.76 | 66750 | 17015.63 | 223790 | 86380.68 |
| Hypoxanthine | 140300 | 35551.62 | 153230 | 50274.26 | 444310 | 107383.4 |
| Inosine | 13372000 | 2035698 | 8796000 | 1068142 | 12793000 | 1406024 |
| Inosinic acid | 1022700 | 114663.9 | 1526900 | 105724.2 | 1170400 | 59812.15 |
| Leucine | 80740 | 16728.75 | 62560 | 19009.19 | 51550 | 5623.428 |
| Lysine | 12204 | 3349.371 | 9921.1 | 4214.313 | 5126 | 1039.707 |
| Malonate | 16525 | 1982.132 | 19890 | 2376.293 | 16880 | 1491.368 |
| Methionine | 109820 | 22289.11 | 88350 | 30557.99 | 68980 | 11635.51 |
| Ophthalmic acid | 37560 | 10077.58 | 18015 | 6646.376 | 12049 | 1874.918 |
| Ornithine | 59465 | 14579.87 | 147860 | 33448.18 | 50481 | 17945.02 |
| Pantothenic acid | 463600 | 39442 | 449000 | 44371.91 | 396000 | 24423.12 |
| Phenylalanine | 110610 | 20771.67 | 88320 | 30500.53 | 65250 | 7365.313 |
| Pterin | 46620 | 16783.01 | 55883 | 18478.41 | 14746.4 | 4737.955 |
| Taurine | 75280000 | 4377056 | 70280000 | 5203926 | 68570000 | 3707951 |
| Threonine | 837700 | 165664.7 | 664000 | 162817.4 | 702800 | 130316.9 |
| Tryptophan | 20801 | 2854.626 | 19700 | 2722.825 | 19200 | 1548.404 |
| Tyrosine | 137870 | 25104.7 | 112180 | 25019.17 | 91860 | 10704.87 |
| UMP | 592500 | 80291.45 | 587000 | 45284.29 | 559900 | 49745.9 |
| Uric acid | 2330100 | 512041.2 | 734650 | 232918.1 | 1672000 | 276209.5 |
| Uridine | 940800 | 162441.7 | 332400 | 66555.61 | 977400 | 135392.6 |
| Valine | 29470 | 5941.961 | 22720 | 6793.801 | 18993 | 2345.313 |
| Xanthine | 419360 | 91839.7 | 415410 | 109980.9 | 1075300 | 166988 |

**Table S2.** Pathway analysis (MetPa) results conducted on the concentration levels of the VIP>1 metabolites identified for the Regressed/ Mid PLS-DA model. Global test and Relative-betweeness Centrality were selected as algorithms for QEA and topological analysis respectively. Zebrafish (*Danio rerio*) KEGG pathway library was used as reference.

| **Regressed/ Mid** | | | | | |
| --- | --- | --- | --- | --- | --- |
| Metabolic pathway | Total compounds | Hits | p-value | FDR | Impact |
| Purine metabolism | 66 | 8 | 3.92 x 10^-5^ | 0.0006 | 0.26 |
| Ala, Asp and Glu metabolism | 27 | 3 | 4.63 x 10^-5^ | 0.0006 | 0.22 |
| Pyrimidine metabolism | 41 | 2 | 0.0006 | 0.0047 | 0.02 |
| TCA cycle | 20 | 1 | 0.0013 | 0.0067 | 0.03 |
| Pyruvate metabolism | 22 | 1 | 0.0013 | 0.0067 | 0 |
| Taurine and hypotaurine metabolism | 7 | 1 | 0.0062 | 0.0257 | 0.4 |
| Arginine biosynthesis | 14 | 3 | 0.0115 | 0.0409 | 0.08 |
| Tyrosine metabolism | 42 | 3 | 0.0132 | 0.0413 | 0.16 |
| Lysine degradation | 25 | 2 | 0.0251 | 0.0698 | 0.13 |
| Cys and Met metabolism | 33 | 2 | 0.0320 | 0.0801 | 0.10 |
| Ketone bodies metabolism | 5 | 2 | 0.0430 | 0.0896 | 0.6 |
| Butanoate metabolism | 15 | 2 | 0.0430 | 0.0896 | 0.11 |
| Histidine metabolism | 15 | 1 | 0.0764 | 0.1273 | 0 |
| beta-Alanine metabolism | 18 | 1 | 0.0764 | 0.1273 | 0 |
| Aminoacyl-tRNA biosynthesis | 48 | 10 | 0.0853 | 0.1291 | 0 |
| Val, Leu and Ile degradation | 40 | 3 | 0.0878 | 0.1291 | 0 |
| Biotin metabolism | 10 | 1 | 0.1596 | 0.2143 | 0 |
| Arg and Pro metabolism | 38 | 1 | 0.1629 | 0.2143 | 0.05 |
| Pantothenate and CoA biosynthesis | 18 | 1 | 0.1913 | 0.2169 | 0 |
| Phe, Tyr and Trp biosynthesis | 4 | 2 | 0.1916 | 0.2169 | 1 |
| Phenylalanine metabolism | 8 | 2 | 0.1916 | 0.2169 | 0.36 |
| Val, Leu and Ile degradation | 8 | 3 | 0.1995 | 0.2169 | 0 |
| Gly, Ser and Thr metabolism | 33 | 1 | 0.2271 | 0.2365 | 0.02 |

**Table S3.** Pathway analysis (MetPa) results conducted on the concentration levels of the VIP>1 metabolites identified for the Mid/ Late PLS-DA model. Global test and Relative-betweeness Centrality were selected as algorithms for QEA and topological analysis respectively. Zebrafish (*Danio rerio*) KEGG pathway library was used as reference.

| **Mid/ Late** | | | | | |
| --- | --- | --- | --- | --- | --- |
| Metabolic pathway | Total compounds | Hits | p-value | FDR | Impact |
| Pyrimidine metabolism | 41 | 3 | 1.88 x 10^-5^ | 0.0003 | 0.11 |
| Ala, Asp and Glu metabolism | 27 | 3 | 3.73 x 10^-5^ | 0.0003 | 0.23 |
| Histidine metabolism | 15 | 2 | 8.09 x 10^-5^ | 0.0003 | 0.22 |
| Aminoacyl-tRNA biosynthesis | 48 | 4 | 8.16 x 10^-5^ | 0.0003 | 0 |
| Galactose metabolism | 27 | 3 | 9.61 x 10^-5^ | 0.0003 | 0.13 |
| Arg and Pro metabolism | 38 | 3 | 9.90 x 10^-5^ | 0.0003 | 0.22 |
| Gln and Glu metabolism | 6 | 1 | 0.0001 | 0.0003 | 0.5 |
| Nucleotide sugar metabolism | 39 | 2 | 0.0002 | 0.0005 | 0.02 |
| Glutathione metabolism | 28 | 3 | 0.0003 | 0.0008 | 0.28 |
| Glycerophospholipid metabolism | 38 | 1 | 0.0004 | 0.0008 | 0.04 |
| Sphingolipid metabolism | 21 | 1 | 0.0004 | 0.0008 | 0.01 |
| Butanoate metabolism | 15 | 2 | 0.0004 | 0.0008 | 0.11 |
| Taurine and hypotaurine metabolism | 7 | 1 | 0.0005 | 0.0008 | 0.2 |
| Arginine biosynthesis | 14 | 3 | 0.0016 | 0.0026 | 0.41 |
| Purine metabolism | 66 | 7 | 0.0016 | 0.0026 | 0.32 |
| Fatty acid biosynthesis | 47 | 1 | 0.0088 | 0.0127 | 0 |
| Starch and sucrose metabolism | 17 | 1 | 0.0124 | 0.0147 | 0.39 |
| Ketone bodies metabolism | 5 | 1 | 0.0127 | 0.0147 | 0.6 |
| Val, Leu and Ile degradation | 40 | 1 | 0.0127 | 0.0147 | 0 |
| Tyrosine metabolism | 42 | 1 | 0.0127 | 0.0147 | 0 |
| Lysine degradation | 25 | 1 | 0.0203 | 0.0226 | 0.13 |
| Gly, Ser and Thr metabolism | 33 | 1 | 0.0238 | 0.0256 | 0 |
| beta-Alanine metabolism | 18 | 1 | 0.0322 | 0.0323 | 0 |

**Table S4.** Pathway analysis (MetPa) results conducted on the concentration levels of the VIP>1 metabolites identified for the Late/ Regressed PLS-Da model. Global test and Relative-betweeness Centrality were selected as algorithms for QEA and topological analysis respectively. Zebrafish (*Danio rerio*) KEGG pathway library was used as reference.

| **Late/ Regressed** | | | | | |
| --- | --- | --- | --- | --- | --- |
| Metabolic pathway | Total compounds | Hits | p-value | FDR | Impact |
| Arginine biosynthesis | 14 | 3 | 5.94 x 10^-7^ | 1.88 x 10^-5^ | 0.19 |
| Arg and Pro metabolism | 38 | 3 | 1.05 x 10^-6^ | 1.88 x 10^-5^ | 0.15 |
| Cys and Met metabolism | 33 | 2 | 2.53 x 10^-6^ | 3.04 x 10^-5^ | 0.10 |
| Glutathione metabolism | 28 | 2 | 5.08 x 10^-6^ | 4.56 x 10^-5^ | 0.28 |
| Taurine and hypotaurine metabolism | 7 | 1w | 6.33 x 10^-6^ | 4.56 x 10^-5^ | 0.2 |
| Histidine metabolism | 15 | 2 | 9.47 x 10^-6^ | 5.68 x 10^-5^ | 0.22 |
| Val, Leu and Ile degradation | 40 | 3 | 1.25 x 10^-5^ | 6.44 x 10^-5^ | 0 |
| Tyrosine metabolism | 42 | 3 | 1.76 x 10^-5^ | 7.91 x 10^-5^ | 0.16 |
| Butanoate metabolism | 15 | 2 | 3.26 x 10^-5^ | 0.0001 | 0.11 |
| Pantothenate and CoA biosynthesis | 18 | 2 | 5.76 x 10^-5^ | 0.0002 | 0 |
| Ketone bodies metabolism | 5 | 1 | 0.0001 | 0.0004 | 0.6 |
| beta-Alanine metabolism | 18 | 1 | 0.0002 | 0.0006 | 0 |
| Ala, Asp and Glu metabolism | 27 | 3 | 0.0002 | 0.0006 | 0.23 |
| Aminoacyl-tRNA biosynthesis | 48 | 12 | 0.0002 | 0.0006 | 0 |
| Phe, Tyr and Trp biosynthesis | 4 | 2 | 0.0007 | 0.0016 | 1 |
| Phenylalanine metabolism | 8 | 2 | 0.0007 | 0.0016 | 0.36 |
| Galactose metabolism | 27 | 2 | 0.0009 | 0.0018 | 0.07 |
| Starch and sucrose metabolism | 17 | 1 | 0.0010 | 0.0019 | 0.39 |
| Lysine degradation | 25 | 1 | 0.0015 | 0.0022 | 0 |
| Biotin metabolism | 10 | 1 | 0.0015 | 0.0022 | 0 |
| Gln and Glu metabolism | 6 | 1 | 0.0017 | 0.0022 | 0.5 |
| Nucleotide sugar metabolism | 39 | 2 | 0.0019 | 0.0024 | 0.02 |
| Gly, Ser and Thr metabolism | 33 | 2 | 0.0025 | 0.0031 | 0.02 |
| Purine metabolism | 66 | 4 | 0.0041 | 0.0047 | 0.09 |
| Tryptophan metabolism | 39 | 1 | 0.0098 | 0.0110 | 0.14 |

**Table S5.** Multiple t-test results conducted on the VIP>1 metabolites identified for each PLS-DA model.

| **Regressed/ Mid** | | | **Mid/ Late** | | | **Late/ Regressed** | | |
| --- | --- | --- | --- | --- | --- | --- | --- | --- |
| **VIP metabolite** | **p-value** | **FDR** | **VIP metabolite** | **p-value** | **FDR** | **VIP metabolite** | **p-value** | **FDR** |
| Asparagine | 3.15 x 10^-5^ | 0.0009 | Inosinic acid | 1.64 x 10^-5^ | 0.0004 | Taurine | 6.33 x 10^-6^ | 0.0002 |
| Uridine | 8.64 x 10^-5^ | 0.0013 | CMP | 2.69 x 10^-5^ | 0.0004 | Ophthalmic acid | 2.32 x 10^-5^ | 0.0003 |
| Uric acid | 0.0004 | 0.0040 | GMP | 3.66 x 10^-5^ | 0.0004 | Glutathione | 4.98 x 10^-5^ | 0.0004 |
| Adenine | 0.0008 | 0.0062 | Glutamic acid | 0.0001 | 0.0007 | Arginine | 5.79 x 10^-5^ | 0.0004 |
| Glycogen | 0.0013 | 0.0081 | Acetylglutamine | 0.0001 | 0.0007 | Acetoacetic acid | 0.0001 | 0.0008 |
| Guanosine | 0.0059 | 0.0206 | EAP | 0.0004 | 0.0018 | Histidine | 0.0002 | 0.0009 |
| Adenosine | 0.0061 | 0.0206 | Glucose 1-phosphate | 0.0004 | 0.0018 | Phenylalanine | 0.0005 | 0.0020 |
| Hypotaurine | 0.0062 | 0.0206 | Taurine | 0.0005 | 0.0018 | Pantothenic acid | 0.0006 | 0.0023 |
| GMP | 0.0062 | 0.0206 | AMP | 0.0011 | 0.0038 | Glucose | 0.0010 | 0.0032 |
| Aminoadipic acid | 0.0081 | 0.0237 | Asparagine | 0.0012 | 0.0038 | Ciliatine | 0.0011 | 0.0032 |
| Acetylglutamine | 0.0087 | 0.0237 | Uridine | 0.0017 | 0.0049 | Lysine | 0.0015 | 0.0038 |
| Ophthalmic acid | 0.0104 | 0.0260 | Allantoin | 0.0021 | 0.0051 | Tyrosine | 0.0015 | 0.0038 |
| Inosine | 0.0204 | 0.0470 | Pantothenic acid | 0.0021 | 0.0051 | Glutamic acid | 0.0017 | 0.0039 |
| Adipic acid | 0.0299 | 0.0640 | Pterin | 0.0048 | 0.0107 | Valine | 0.0020 | 0.0044 |
| Inosinic acid | 0.0329 | 0.0658 | Ornithine | 0.0057 | 0.0110 | Leucine | 0.0024 | 0.0048 |
| Allantoin | 0.0390 | 0.0731 | Citrulline | 0.0057 | 0.0110 | Methionine | 0.0044 | 0.0080 |
| CMP | 0.0443 | 0.0782 | Malonate | 0.0088 | 0.0160 | Fructose | 0.0045 | 0.0080 |
| 3-Hydroxybutyric acid | 0.0530 | 0.0883 | Glucose | 0.0124 | 0.0207 | Azelaic acid | 0.0060 | 0.0100 |
| Acetoacetic acid | 0.0685 | 0.1082 | Acetoacetic acid | 0.0127 | 0.0207 | Tryptophan | 0.0098 | 0.0154 |
| Aspartic acid | 0.0764 | 0.1145 | Fumaric acid | 0.0135 | 0.0209 | Glucose 1-phosphate | 0.0103 | 0.0154 |
| AMP | 0.1461 | 0.2004 | UMP | 0.0169 | 0.0249 | Guanosine | 0.0132 | 0.0189 |
| Phenylalanine | 0.1470 | 0.2004 | Xanthine | 0.0192 | 0.0262 | GMP | 0.0147 | 0.0195 |
| Lysine | 0.1596 | 0.2036 | Adenine | 0.0201 | 0.0262 | Uric acid | 0.0150 | 0.0195 |
| Arginine | 0.1629 | 0.2036 | Aminoadipic acid | 0.0203 | 0.0262 | Creatine | 0.0159 | 0.0195 |
| Valine | 0.1913 | 0.2191 | Fructose | 0.0216 | 0.0268 | Alanine | 0.0165 | 0.0195 |
| Leucine | 0.1963 | 0.2191 | Glutathione | 0.0233 | 0.0273 | UMP | 0.0169 | 0.0195 |
| Methionine | 0.1972 | 0.2191 | Creatine | 0.0238 | 0.0273 | Glycogen | 0.0240 | 0.0267 |
| Homoserine | 0.2271 | 0.2349 | Uric acid | 0.0289 | 0.0320 | Homoserine | 0.0291 | 0.0301 |
| Threonine | 0.2271 | 0.2349 | Histidine | 0.0322 | 0.0333 | Threonine | 0.0291 | 0.0301 |
| Tyrosine | 0.2831 | 0.2831 | Alanine | 0.0323 | 0.0333 | Inosine | 0.0366 | 0.0366 |
|  |  |  | Hypoxanthine | 0.0613 | 0.0613 |  |  |  |

**Table S6.** One-way ANOVA results conducted on the VIP>1 metabolites identified in the three PLS-DA models investigating the effect of GnRH in the investigated reproductive stages (Regressed, Mid and Late). Tukey’s post hoc test was used as multiple comparison statistical test following ANOVA. P-value <0.05 indicate statistically significant differences between groups. False Discovery Rate (FDR) indicate the p-value adjusted for multiple comparison.

| **GnRH treatments (Ctrl/ PBS+GnRH/ GnRH+GnRH)** | | | | | | | | |
| --- | --- | --- | --- | --- | --- | --- | --- | --- |
| **Regressed** | | | **Mid** | | | **Late** | | |
| **VIP metabolites** | **p-value** | **FDR** | **VIP metabolites** | **p-value** | **FDR** | **VIP metabolites** | **p-value** | **FDR** |
| Guanosine | 0.00025 | 0.0064 | Acetoacetic acid | 0.0031 | 0.0203 | AMP | 0.0027 | 0.0653 |
| Adenosine | 0.00065 | 0.0085 | Ribose 5-phosphate | 0.0035 | 0.0203 | Pimelic acid | 0.0086 | 0.1029 |
| Uridine | 0.0057 | 0.0381 | Aspartic acid | 0.0042 | 0.0203 | Succinic acid | 0.0191 | 0.1529 |
| 4-Hydroxybenzaldehyde | 0.0059 | 0.0381 | Itaconic acid | 0.0046 | 0.0203 | Oligomaltose | 0.0740 | 0.2332 |
| Cytidine | 0.0078 | 0.0393 | Taurine | 0.0048 | 0.0203 | Aspartic acid | 0.0759 | 0.2332 |
| Inosine | 0.0107 | 0.0393 | UDP-GlcNAc | 0.0049 | 0.0203 | Raffinose | 0.0963 | 0.2332 |
| Hypoxanthine | 0.0119 | 0.0393 | EAP | 0.0087 | 0.0275 | Fructose | 0.0992 | 0.2332 |
| Xanthine | 0.0121 | 0.0393 | DHAP | 0.0088 | 0.0275 | Citrulline | 0.1043 | 0.2332 |
| Glucose | 0.0167 | 0.0438 | Sucrose | 0.0154 | 0.0426 | Adenine | 0.1181 | 0.2332 |
| Uric acid | 0.0168 | 0.0438 | Adenine | 0.0184 | 0.0460 | DHAP | 0.1236 | 0.2332 |
| Fructose | 0.0190 | 0.0449 | Serine | 0.0260 | 0.0592 | Monomethyl glutarate | 0.1275 | 0.2332 |
| Sucrose | 0.0347 | 0.0751 | Adenosine | 0.0320 | 0.0618 | Glucose | 0.1311 | 0.2332 |
| Glutamine | 0.0391 | 0.0782 | Allantoin | 0.0321 | 0.0618 | Acetoacetic acid | 0.1313 | 0.2332 |
| GMP | 0.0605 | 0.0980 | CMP | 0.0487 | 0.0869 | UDP-GlcNAc | 0.1360 | 0.2332 |
| Oligomaltose | 0.0632 | 0.0980 | Pantothenic acid | 0.0539 | 0.0899 | Glycogen | 0.1552 | 0.2483 |
| Itaconic acid | 0.0640 | 0.0980 | Guanosine | 0.0587 | 0.0917 | NMDA | 0.1850 | 0.2774 |
| Glucose 6-phosphate | 0.0664 | 0.0980 | Asparagine | 0.0631 | 0.0928 | Hippuric acid | 0.2452 | 0.3462 |
| Glycogen | 0.0679 | 0.0980 | Hypoxanthine | 0.0770 | 0.1070 | Methionine | 0.2651 | 0.3534 |
| 3-Hydroxybutyric acid | 0.0762 | 0.1043 | Xanthine | 0.0864 | 0.1138 | Taurine | 0.3112 | 0.3895 |
| Ribose 5-phosphate | 0.0886 | 0.11 | Glutamine | 0.0953 | 0.1177 | Adenosine | 0.3383 | 0.3895 |
| Glutaric acid | 0.0889 | 0.11 | Uridine | 0.0993 | 0.1177 | Glycerol 3-phosphate | 0.3630 | 0.3895 |
| Monomethyl glutarate | 0.0935 | 0.1105 | 3-Hydroxybutyric acid | 0.1036 | 0.1177 | Sucrose | 0.3725 | 0.3895 |
| Raffinose | 0.1084 | 0.1226 | 4-Hydroxybenzaldehyde | 0.1299 | 0.1412 | Aminoadipic acid | 0.3733 | 0.3895 |
| Cystathionine | 0.1482 | 0.1606 | Azelaic acid | 0.1529 | 0.1592 | Histidine | 0.3997 | 0.3997 |
| NMDA | 0.1821 | 0.1894 | Hippuric acid | 0.1839 | 0.1839 |  |  |  |
| UDP-GlcNAc | 0.2301 | 0.2301 |  |  |  |  |  |  |

**Table S7.** Multiple t-test results conducted on the VIP>1 metabolites identified in the PLS-DA model investigating the effect of GnRH during the regressed gonadal phase. P-value <0.05 indicate statistically significant differences between groups. False Discovery Rate (FDR) indicate the p-value adjusted for multiple comparison.

| **Regressed - Ctrl/ PBS+GnRH** | | | **Regressed - Ctrl/ GnRH+GnRH** | | |
| --- | --- | --- | --- | --- | --- |
| **VIP metabolite** | **p-value** | **FDR** | **VIP metabolite** | **p-value** | **FDR** |
| Adenosine | 5.81E-05 | 0.0015 | Adenosine | 0.0425 | 0.5840 |
| Guanosine | 0.0006 | 0.0083 | Guanosine | 0.0449 | 0.5840 |
| 4-Hydroxybenzaldehyde | 0.0055 | 0.0399 | Cytidine | 0.1105 | 0.6798 |
| Uridine | 0.0061 | 0.0399 | 3-Hydroxybutyric acid | 0.1322 | 0.6798 |
| Hypoxanthine | 0.0091 | 0.0446 | Glutamine | 0.1446 | 0.6798 |
| Cytidine | 0.0106 | 0.0446 | Ribose 5-phosphate | 0.1893 | 0.6798 |
| Xanthine | 0.0120 | 0.0446 | Sucrose | 0.2058 | 0.6798 |
| 3-Hydroxybutyric acid | 0.0175 | 0.0544 | Itaconic acid | 0.2270 | 0.6798 |
| Uric acid | 0.0203 | 0.0544 | Glucose 6-phosphate | 0.2353 | 0.6798 |
| Inosine | 0.0209 | 0.0544 | Maltose | 0.3405 | 0.6907 |
| Sucrose | 0.0395 | 0.0809 | Stachyose | 0.3490 | 0.6907 |
| Glucose | 0.0414 | 0.0809 | Xanthine | 0.3533 | 0.6907 |
| Fructose | 0.0435 | 0.0809 | Uridine | 0.3637 | 0.6907 |
| Itaconic acid | 0.0436 | 0.0809 | Raffinose | 0.3719 | 0.6907 |
| Cystathionine | 0.0591 | 0.1024 | Methylglutarate | 0.4562 | 0.7907 |
| Maltose | 0.0634 | 0.1024 | Hypoxanthine | 0.5678 | 0.8638 |
| Glycogen | 0.0670 | 0.1024 | 4-Hydroxybenzaldehyde | 0.5745 | 0.8638 |
| GMP | 0.0802 | 0.1158 | Inosine | 0.5980 | 0.8638 |
| Raffinose | 0.1050 | 0.1437 | Glutaric acid | 0.6545 | 0.8766 |
| UDP-GlcNAc | 0.1269 | 0.1512 | GMP | 0.7049 | 0.8766 |
| NMDA | 0.1283 | 0.1512 | UDP-GlcNAc | 0.7155 | 0.8766 |
| Methylglutarate | 0.1318 | 0.1512 | Fructose | 0.7709 | 0.8766 |
| Glutaric acid | 0.1338 | 0.1512 | Cystathionine | 0.7977 | 0.8766 |
| Glucose 6-phosphate | 0.1554 | 0.1684 | Glucose | 0.8100 | 0.8766 |
| Glutamine | 0.2830 | 0.2939 | Uric acid | 0.8429 | 0.8766 |
| Ribose 5-phosphate | 0.2939 | 0.2939 | NMDA | 0.9656 | 0.9656 |

**Table S8.** Multiple t-test results conducted on the VIP>1 metabolites identified in the PLS-DA model investigating the effect of GnRH during mid recrudescence. P-value <0.05 indicate statistically significant differences between groups. False Discovery Rate (FDR) indicate the p-value adjusted for multiple comparison.

| **Mid - Ctrl/ PBS+GnRH** | | | **Mid - Ctrl/ GnRH+GnRH** | | |
| --- | --- | --- | --- | --- | --- |
| **VIP metabolite** | **p-value** | **FDR** | **VIP metabolite** | **p-value** | **FDR** |
| DHAP | 0.0011 | 0.0273 | Acetoacetic acid | 0.0142 | 0.2531 |
| Aspartic acid | 0.0038 | 0.0471 | Taurine | 0.0297 | 0.2531 |
| UDP-GlcNAc | 0.0100 | 0.0831 | Ribose 5-phosphate | 0.0397 | 0.2531 |
| Allantoin | 0.0255 | 0.1592 | Sucrose | 0.0405 | 0.2531 |
| EAP | 0.0431 | 0.2077 | Itaconic acid | 0.0620 | 0.3098 |
| Hypoxanthine | 0.0531 | 0.2077 | Serine | 0.0806 | 0.3357 |
| Asparagine | 0.0624 | 0.2077 | Adenine | 0.1273 | 0.4448 |
| CMP | 0.0747 | 0.2077 | Adenosine | 0.1423 | 0.4448 |
| Glutamine | 0.0926 | 0.2077 | 4-Hydroxybenzaldehyde | 0.1913 | 0.5314 |
| Ribose 5-phosphate | 0.1051 | 0.2077 | Guanosine | 0.2269 | 0.5672 |
| Itaconic acid | 0.1079 | 0.2077 | Aspartic acid | 0.3139 | 0.7133 |
| Xanthine | 0.1148 | 0.2077 | EAP | 0.4055 | 0.8185 |
| Uridine | 0.1195 | 0.2077 | DHAP | 0.4256 | 0.8185 |
| Pantothenic acid | 0.1239 | 0.2077 | Pantothenic acid | 0.5107 | 0.8880 |
| 3-Hydroxybutyric acid | 0.1254 | 0.2077 | Uridine | 0.5328 | 0.8880 |
| Adenine | 0.1467 | 0.2077 | 3-Hydroxybutyric acid | 0.5987 | 0.8987 |
| Azelaic acid | 0.1556 | 0.2077 | Xanthine | 0.6243 | 0.8987 |
| Taurine | 0.1557 | 0.2077 | CMP | 0.6579 | 0.8987 |
| Hippuric acid | 0.1605 | 0.2077 | Glutamine | 0.7298 | 0.8987 |
| Adenosine | 0.1661 | 0.2077 | Azelaic acid | 0.7390 | 0.8987 |
| Guanosine | 0.1954 | 0.2326 | UDP-GlcNAc | 0.7549 | 0.8987 |
| Serine | 0.2590 | 0.2943 | Hippuric acid | 0.8090 | 0.9194 |
| 4-Hydroxybenzaldehyde | 0.4468 | 0.4856 | Allantoin | 0.9102 | 0.9539 |
| Sucrose | 0.5779 | 0.6019 | Hypoxanthine | 0.9158 | 0.9539 |
| Acetoacetic acid | 0.6408 | 0.6408 | Asparagine | 0.9671 | 0.9671 |

**Table S9.** Multiple t-test results conducted on the VIP>1 metabolites identified in the PLS-DA model investigating the effect of GnRH during late recrudescence. P-value <0.05 indicate statistically significant differences between groups. False Discovery Rate (FDR) indicate the p-value adjusted for multiple comparison.

| **Late - Ctrl/ PBS+GnRH** | | | **Late - Ctrl/ GnRH+GnRH** | | |
| --- | --- | --- | --- | --- | --- |
| **VIP metabolite** | **p-value** | **FDR** | **VIP metabolite** | **p-value** | **FDR** |
| Succinic acid | 0.0006 | 0.0146 | Pimelic acid | 0.0027 | 0.0372 |
| AMP | 0.0243 | 0.1221 | AMP | 0.0031 | 0.0372 |
| DHAP | 0.0274 | 0.1221 | Aspartic acid | 0.0227 | 0.1813 |
| UDP-GlcNAc | 0.0290 | 0.1221 | Acetoacetic acid | 0.0519 | 0.2882 |
| Maltose | 0.0319 | 0.1221 | Adenine | 0.0601 | 0.2882 |
| Raffinose | 0.0350 | 0.1221 | Fructose | 0.0809 | 0.2913 |
| Glycogen | 0.0384 | 0.1221 | Maltose | 0.1068 | 0.2913 |
| Pimelic acid | 0.0407 | 0.1221 | DHAP | 0.1099 | 0.2913 |
| Methylglutarate | 0.0514 | 0.1328 | Raffinose | 0.1165 | 0.2913 |
| NMDA | 0.0553 | 0.1328 | Citrulline | 0.1214 | 0.2913 |
| Citrulline | 0.0724 | 0.1581 | Glucose | 0.1366 | 0.2980 |
| Fructose | 0.0816 | 0.1632 | Methionine | 0.1635 | 0.3216 |
| Glucose | 0.0962 | 0.1776 | Sucrose | 0.1742 | 0.3216 |
| Adenosine | 0.1121 | 0.1905 | NMDA | 0.2188 | 0.3751 |
| Glycerol 3-phosphate | 0.1191 | 0.1905 | Stachyose | 0.2562 | 0.3814 |
| Adenine | 0.1502 | 0.2050 | Aminoadipic acid | 0.2664 | 0.3814 |
| Histidine | 0.1537 | 0.2050 | Adenosine | 0.2787 | 0.3814 |
| Hippuric acid | 0.1537 | 0.2050 | Methylglutarate | 0.2861 | 0.3814 |
| Taurine | 0.1665 | 0.2103 | Succinic acid | 0.3684 | 0.4430 |
| Sucrose | 0.1787 | 0.2144 | Glycerol 3-phosphate | 0.3718 | 0.4430 |
| Aspartic acid | 0.2614 | 0.2921 | Taurine | 0.3996 | 0.4430 |
| Methionine | 0.2697 | 0.2921 | Histidine | 0.4061 | 0.4430 |
| Aminoadipic acid | 0.2799 | 0.2921 | UDP-GlcNAc | 0.4726 | 0.4921 |
| Acetoacetic acid | 0.3557 | 0.3557 | Hippuric acid | 0.4921 | 0.4921 |

**Table S10.** One-way ANOVA results conducted on the VIP>1 metabolites identified in the three PLS-DA models investigating the effect of GnIH in the investigated reproductive stages (Regressed, Mid and Late). Tukey’s post hoc test was used as multiple comparison statistical test following ANOVA. P-value <0.05 indicate statistically significant differences between groups. False Discovery Rate (FDR) indicate the p-value adjusted for multiple comparison.

| **GnIH treatments (Ctrl/ PBS+GnIH/ GnIH+GnIH)** | | | | | | | | |
| --- | --- | --- | --- | --- | --- | --- | --- | --- |
| **Regressed** | | | **Mid** | | | **Late** | | |
| **VIP metabolites** | **p-value** | **FDR** | **VIP metabolites** | **p-value** | **FDR** | **VIP metabolites** | **p-value** | **FDR** |
| Citrulline | 0.0161 | 0.1717 | Hippuric acid | 0.0353 | 0.3307 | Allantoin | 0.0011 | 0.0196 |
| Itaconic acid | 0.0178 | 0.1717 | Pantothenic acid | 0.0642 | 0.3307 | CMP | 0.0070 | 0.0630 |
| Sucrose | 0.0290 | 0.1717 | Creatine | 0.0876 | 0.3307 | Pimelic acid | 0.0142 | 0.0852 |
| N-Acetylglutamate | 0.0455 | 0.1717 | Alanine | 0.0990 | 0.3307 | Fumaric acid | 0.0265 | 0.1129 |
| Pterin | 0.0651 | 0.1717 | Asparagine | 0.1115 | 0.3307 | EAP | 0.0313 | 0.1129 |
| Glutaric acid | 0.0682 | 0.1717 | Glutamine | 0.1240 | 0.3307 | Succinic acid | 0.0446 | 0.1337 |
| Alanine | 0.0726 | 0.1717 | Glucose 6-phosphate | 0.1854 | 0.4238 | Aspartic acid | 0.0537 | 0.1380 |
| Uric acid | 0.0777 | 0.1717 | Adenine | 0.2588 | 0.4257 | Uridine | 0.0782 | 0.1760 |
| Tyrosine | 0.0852 | 0.1717 | Inosinic acid | 0.2692 | 0.4257 | 3-Hydroxybutyric acid | 0.0903 | 0.1806 |
| Guanosine | 0.0877 | 0.1717 | Inosine | 0.2872 | 0.4257 | Raffinose | 0.1449 | 0.2440 |
| Creatine | 0.0919 | 0.1717 | Guanosine | 0.2927 | 0.4257 | DHA | 0.1652 | 0.2440 |
| Adenine | 0.1070 | 0.1717 | Glucose | 0.3389 | 0.4518 | AMP | 0.1732 | 0.2440 |
| Cytidine | 0.1086 | 0.1717 | Uridine | 0.4622 | 0.5406 | Glycogen | 0.1762 | 0.2440 |
| Glucose 6-phosphate | 0.1105 | 0.1717 | AMP | 0.5063 | 0.5406 | Glutaric acid | 0.2018 | 0.2595 |
| Hypoxanthine | 0.1187 | 0.1717 | Methionine | 0.5068 | 0.5406 | Adipic acid | 0.2440 | 0.2928 |
| Succinic acid | 0.1253 | 0.1717 | Glutamic acid | 0.6195 | 0.6195 | Serine | 0.3101 | 0.3489 |
| Serine | 0.1294 | 0.1717 |  |  |  | Hippuric acid | 0.3538 | 0.3746 |
| Xanthine | 0.1344 | 0.1717 |  |  |  | Alanine | 0.4660 | 0.4660 |
| N-Acetylserine | 0.1601 | 0.1933 |  |  |  |  |  |  |
| GMP | 0.1681 | 0.1933 |  |  |  |  |  |  |
| Glutathione | 0.1869 | 0.2047 |  |  |  |  |  |  |
| Ornithine | 0.2036 | 0.2128 |  |  |  |  |  |  |
| Ribose 5-phosphate | 0.3116 | 0.3116 |  |  |  |  |  |  |

**Table S11.** Multiple t-test results conducted on the VIP>1 metabolites identified in the PLS-DA model investigating the effect of GnIH during the regressed gonadal phase. P-value <0.05 indicate statistically significant differences between groups. False Discovery Rate (FDR) indicate the p-value adjusted for multiple comparison.

| **Regressed - Ctrl/ PBS+GnIH** | | | **Regressed - Ctrl/ GnIH+GnIH** | | |
| --- | --- | --- | --- | --- | --- |
| **VIP metabolite** | **p-value** | **FDR** | **VIP metabolite** | **p-value** | **FDR** |
| Creatine | 0.0163 | 0.1885 | Xanthine | 0.0102 | 0.1098 |
| Alanine | 0.0164 | 0.1885 | Itaconic acid | 0.0120 | 0.1098 |
| Citrulline | 0.0769 | 0.5427 | Hypoxanthine | 0.0275 | 0.1098 |
| Tyrosine | 0.0944 | 0.5427 | Sucrose | 0.0286 | 0.1098 |
| Glucose 6-phosphate | 0.1514 | 0.5759 | Guanosine | 0.0355 | 0.1098 |
| Pterin | 0.1788 | 0.5759 | Cytidine | 0.0386 | 0.1098 |
| Ribose 5-phosphate | 0.2138 | 0.5759 | Uric acid | 0.0391 | 0.1098 |
| Ornithine | 0.2358 | 0.5759 | Adenine | 0.0416 | 0.1098 |
| GMP | 0.2771 | 0.5759 | Glutaric acid | 0.0430 | 0.1098 |
| Succinic acid | 0.3507 | 0.5759 | N-Acetylglutamic acid | 0.0808 | 0.1858 |
| Glutathione | 0.3534 | 0.5759 | Alanine | 0.1716 | 0.3487 |
| Adenine | 0.3591 | 0.5759 | Creatine | 0.1819 | 0.3487 |
| Serine | 0.3683 | 0.5759 | Succinic acid | 0.2370 | 0.3703 |
| Uric acid | 0.3910 | 0.5759 | Citrulline | 0.2383 | 0.3703 |
| Guanosine | 0.3982 | 0.5759 | N-Acetylserine | 0.2415 | 0.3703 |
| Hypoxanthine | 0.4006 | 0.5759 | Serine | 0.2647 | 0.3805 |
| Sucrose | 0.4527 | 0.5917 | Glutathione | 0.3746 | 0.5061 |
| Cytidine | 0.4636 | 0.5917 | Pterin | 0.3961 | 0.5061 |
| N-Acetylserine | 0.4888 | 0.5917 | Glucose 6-phosphate | 0.4383 | 0.5127 |
| N-Acetylglutamic acid | 0.5277 | 0.6057 | Tyrosine | 0.4458 | 0.5127 |
| Xanthine | 0.5531 | 0.6057 | GMP | 0.5075 | 0.5559 |
| Itaconic acid | 0.5917 | 0.6186 | Ornithine | 0.5512 | 0.5763 |
| Glutaric acid | 0.7869 | 0.7869 | Ribose 5-phosphate | 0.9407 | 0.9407 |

**Table S12.** Multiple t-test results conducted on the VIP>1 metabolites identified in the PLS-DA model investigating the effect of GnIH during mid recrudescence. P-value <0.05 indicate statistically significant differences between groups. False Discovery Rate (FDR) indicate the p-value adjusted for multiple comparison.

| **Regressed - Ctrl/ PBS+GnIH** | | | **Regressed - Ctrl/ GnIH+GnIH** | | |
| --- | --- | --- | --- | --- | --- |
| **VIP metabolite** | **p-value** | **FDR** | **VIP metabolite** | **p-value** | **FDR** |
| Creatine | 0.0502 | 0.4704 | Pantothenic acid | 0.0084 | 0.1346 |
| Alanine | 0.0588 | 0.4704 | Glutamine | 0.0219 | 0.1753 |
| Asparagine | 0.1715 | 0.8313 | Hippuric acid | 0.0634 | 0.2163 |
| Glutamine | 0.3289 | 0.8313 | Asparagine | 0.0738 | 0.2163 |
| Uridine | 0.3962 | 0.8313 | Inosine | 0.0891 | 0.2163 |
| Inosinic acid | 0.4239 | 0.8313 | Glucose 6-phosphate | 0.0919 | 0.2163 |
| Glucose 6-phosphate | 0.4735 | 0.8313 | AMP | 0.0946 | 0.2163 |
| Inosine | 0.4744 | 0.8313 | Guanosine | 0.1124 | 0.2248 |
| Pantothenic acid | 0.5322 | 0.8313 | Adenine | 0.1360 | 0.2288 |
| Glucose | 0.5674 | 0.8313 | Methionine | 0.1569 | 0.2288 |
| Guanosine | 0.5715 | 0.8313 | Inosinic acid | 0.1590 | 0.2288 |
| Adenine | 0.7595 | 0.9020 | Creatine | 0.1918 | 0.2288 |
| Glutamic acid | 0.7805 | 0.9020 | Alanine | 0.1954 | 0.2288 |
| Hippuric acid | 0.8150 | 0.9020 | Uridine | 0.2002 | 0.2288 |
| AMP | 0.8456 | 0.9020 | Glucose | 0.2161 | 0.2305 |
| Methionine | 0.9311 | 0.9311 | Glutamic acid | 0.3725 | 0.3725 |

**Table S13.** Multiple t-test results conducted on the VIP>1 metabolites identified in the PLS-DA model investigating the effect of GnIH during late recrudescence. P-value <0.05 indicate statistically significant differences between groups. False Discovery Rate (FDR) indicate the p-value adjusted for multiple comparison.

| **Regressed - Ctrl/ PBS+GnIH** | | | **Regressed - Ctrl/ GnIH+GnIH** | | |
| --- | --- | --- | --- | --- | --- |
| **VIP metabolite** | **p-value** | **FDR** | **VIP metabolite** | **p-value** | **FDR** |
| Allantoin | 0.0006 | 0.0112 | CMP | 0.0045 | 0.0408 |
| Aspartic acid | 0.0287 | 0.1394 | Pimelic acid | 0.0045 | 0.0408 |
| AMP | 0.0307 | 0.1394 | EAP | 0.0101 | 0.0608 |
| Pimelic acid | 0.0310 | 0.1394 | Fumaric acid | 0.0166 | 0.0670 |
| CMP | 0.0539 | 0.1732 | Docosahexaenoic acid | 0.0186 | 0.0670 |
| Uridine | 0.0577 | 0.1732 | Succinic acid | 0.0277 | 0.0716 |
| Raffinose | 0.1254 | 0.3225 | 3-Hydroxybutyric acid | 0.0279 | 0.0716 |
| Succinic acid | 0.1462 | 0.3289 | Uridine | 0.0484 | 0.0992 |
| Glycogen | 0.1781 | 0.3316 | Serine | 0.0496 | 0.0992 |
| Docosahexaenoic acid | 0.1842 | 0.3316 | Allantoin | 0.0666 | 0.1198 |
| Hippuric acid | 0.2741 | 0.4261 | Raffinose | 0.0783 | 0.1281 |
| 3-Hydroxybutyric acid | 0.2841 | 0.4261 | Glutaric acid | 0.0914 | 0.1370 |
| Alanine | 0.3967 | 0.5300 | Glycogen | 0.1045 | 0.1447 |
| Glutaric acid | 0.4122 | 0.5300 | Hippuric acid | 0.1354 | 0.1714 |
| Adipic acid | 0.5976 | 0.7171 | Adipic acid | 0.1428 | 0.1714 |
| EAP | 0.7757 | 0.8297 | AMP | 0.2047 | 0.2303 |
| Fumaric acid | 0.7836 | 0.8297 | Alanine | 0.2830 | 0.2996 |
| Serine | 0.8491 | 0.8491 | Aspartic acid | 0.4272 | 0.4272 |

Table S14. Pathway analysis (MetPa) results investigating the effect of GnRH in the three reproductive phases. Within each reproductive phase, single and double injection treatment groups were independently compared to the control. Global test and Relative-betweeness Centrality were selected as algorithms for QEA and topological analysis respectively. Zebrafish (*Danio rerio*) KEGG pathway library was used as reference.

| **Regressed** | | | | | |
| --- | --- | --- | --- | --- | --- |
| **CTRL/ PBS+GnRH** | **p-value** | **Impact** | **CTRL/ GnRH+GnRH** | **p-value** | **Impact** |
| Purine metabolism | 2.71 x 10^-7^ | 0.16 | Ketone bodies metabolism | 0.13218 | 0 |
| Ketone bodies metabolism | 0.0174 | 0 | Butanoate metabolism | 0.13218 | 0 |
| Butanoate metabolism | 0.0174 | 0 | Ala, Asp and Glu metabolism | 0.14458 | 0.13296 |
| Pyrimidine metabolism | 0.0257 | 0.01 | Arginine biosynthesis | 0.14458 | 0 |
| Nnucleotide sugar metabolism | 0.0347 | 0.01 | Gln and Glu metabolism | 0.14458 | 0 |
| Starch and sucrose metabolism | 0.0547 | 0.66 | Pyrimidine metabolism | 0.14787 | 0.01611 |
| Cysteine and methionine metabolism | 0.0591 | 0.18 | Purine metabolism | 0.17827 | 0.16725 |
| Gly, Ser and Thr metabolism | 0.0591 | 0 | Pentose phosphate pathway | 0.18934 | 0.20598 |
| Galactose metabolism | 0.0652 | 0.16 | Starch and sucrose metabolism | 0.33925 | 0.66269 |
| Ala, Asp and Glu metabolism | 0.2830 | 0.13 | Galactose metabolism | 0.38966 | 0.16407 |
| Arginine biosynthesis | 0.2830 | 0 | Cysteine and methionine metabolism | 0.79766 | 0.17854 |
| Gln and Glu metabolism | 0.2830 | 0 | Gly, Ser and Thr metabolism | 0.79766 | 0 |
| Pentose phosphate pathway | 0.2939 | 0.2 | Nucleotide sugar metabolism | 0.88261 | 0.01426 |
| **Mid** | | | | | |
| **CTRL/ PBS+GnRH** | **p-value** | **Impact** | **CTRL/ GnRH+GnRH** | **p-value** | **Impact** |
| Glycerophospholipid metabolism | 0.00053 | 0.06 | Val, Leu and Ile degradation | 0.0142 | 0 |
| Glycerolipid metabolism | 0.0010 | 0 | Tyrosine metabolism | 0.0142 | 0 |
| Histidine metabolism | 0.0037 | 0 | Ketone bodies metabolism | 0.0166 | 0.6 |
| beta-Alanine metabolism | 0.0037 | 0 | Butanoate metabolism | 0.0166 | 0.11 |
| Nucleotide sugar metabolism | 0.0099 | 0.01 | Taurine and hypotaurine metabolism | 0.0297 | 0.2 |
| Aminoacyl-tRNA biosynthesis | 0.0246 | 0.17 | Pentose phosphate pathway | 0.0397 | 0.2 |
| Ala, Asp and Glu metabolism | 0.0268 | 0.35 | Starch and sucrose metabolism | 0.0404 | 0.05 |
| Pyrimidine metabolism | 0.0282 | 0.02 | Galactose metabolism | 0.0404 | 0.04 |
| Arginine biosynthesis | 0.0381 | 0 | Histidine metabolism | 0.3138 | 0 |
| Purine metabolism | 0.0421 | 0.08 | beta-Alanine metabolism | 0.3138 | 0 |
| Pentose phosphate pathway | 0.1051 | 0.2 | Purine metabolism | 0.3811 | 0.08 |
| Taurine and hypotaurine metabolism | 0.1556 | 0.2 | Fructose and mannose metabolism | 0.4256 | 0.01 |
| Ketone bodies metabolism | 0.3316 | 0.6 | Glycolysis / Gluconeogenesis | 0.4256 | 0 |
| Butanoate metabolism | 0.3316 | 0.11 | Glycerolipid metabolism | 0.4256 | 0 |
| Starch and sucrose metabolism | 0.5778 | 0.05 | Glycerophospholipid metabolism | 0.4776 | 0.06 |
| Galactose metabolism | 0.5778 | 0.04 | Aminoacyl-tRNA biosynthesis | 0.5035 | 0.17 |
| Val, Leu and Ile degradation | 0.6408 | 0 | Arginine biosynthesis | 0.7013 | 0 |
| Tyrosine metabolism | 0.6408 | 0 | Ala, Asp and Glu metabolism | 0.7510 | 0.35 |
| **Late** | | | | | |
| **CTRL/ PBS+GnRH** | **p-value** | **Impact** | **CTRL/ GnRH+GnRH** | **p-value** | **Impact** |
| TCA cycle | 0.00061 | 0.03 | Ala, Asp and Glu metabolism | 0.0354 | 0.22 |
| Nucleotide sugar metabolism | 0.0258 | 0.01 | Arginine biosynthesis | 0.0460 | 0.23 |
| Fructose and mannose metabolism | 0.0274 | 0.01 | Ketone bodies metabolism | 0.0518 | 0.6 |
| Glycolysis / Gluconeogenesis | 0.0274 | 0 | Val, Leu and Ile degradation | 0.0518 | 0 |
| Ala, Asp and Glu metabolism | 0.0331 | 0.21 | Tyrosine metabolism | 0.0518 | 0 |
| Glycerophospholipid metabolism | 0.0682 | 0.09 | Butanoate metabolism | 0.0519 | 0.11 |
| Glycerolipid metabolism | 0.0682 | 0.04 | Aminoacyl-tRNA biosynthesis | 0.0614 | 0 |
| Arginine biosynthesis | 0.0699 | 0.23 | Purine metabolism | 0.0639 | 0.06 |
| Purine metabolism | 0.0784 | 0.06 | Histidine metabolism | 0.0693 | 0.22 |
| Butanoate metabolism | 0.1173 | 0.11 | beta-Alanine metabolism | 0.0693 | 0 |
| Galactose metabolism | 0.1178 | 0.16 | Fructose and mannose metabolism | 0.1099 | 0.01 |
| Starch and sucrose metabolism | 0.123 | 0.52 | Glycolysis / Gluconeogenesis | 0.1099 | 0 |
| Taurine and hypotaurine metabolism | 0.1665 | 0.2 | Starch and sucrose metabolism | 0.1476 | 0.52 |

Table S15. Pathway analysis (MetPa) results investigating the effect of GnIH in the three reproductive phases. Within each reproductive phase, single and double injection treatment groups were independently compared to the control. Global test and Relative-betweeness Centrality were selected as algorithms for QEA and topological analysis respectively. Zebrafish (*Danio rerio*) KEGG pathway library was used as reference.

| **Regressed** | | | | | |
| --- | --- | --- | --- | --- | --- |
| **CTRL/ PBS+GnIH** | **p-value** | **Impact** | **CTRL/ GnIH+GnIH** | **p-value** | **Impact** |
| Aminoacyl-tRNA biosynthesis | 0.0306 | 0.17 | Purine metabolism | 0.0009 | 0.17 |
| Ala, Asp and Glu metabolism | 0.0310 | 0 | Galactose metabolism | 0.0286 | 0.04 |
| Gly, Ser and Thr metabolism | 0.0358 | 0.19 | Starch and sucrose metabolism | 0.0522 | 0.19 |
| Arginine and proline metabolism | 0.0628 | 0.14 | Ala, Asp and Glu metabolism | 0.1733 | 0 |
| Phe, Tyr and Trp biosynthesis | 0.0944 | 0.5 | Arginine biosynthesis | 0.2148 | 0.29 |
| Tyrosine metabolism | 0.0944 | 0.14 | Gly, Ser and Thr metabolism | 0.2188 | 0.19 |
| Arginine biosynthesis | 0.1445 | 0.29 | TCA cycle | 0.2370 | 0.03 |
| Starch and sucrose metabolism | 0.2016 | 0.19 | Aminoacyl-tRNA biosynthesis | 0.2793 | 0.17 |
| Pentose phosphate pathway | 0.2138 | 0.21 | Arginine and proline metabolism | 0.3981 | 0.14 |
| Glutathione metabolism | 0.2604 | 0.26 | Phe, Tyr and Trp biosynthesis | 0.4458 | 0.5 |
| TCA cycle | 0.3507 | 0.03 | Tyrosine metabolism | 0.4458 | 0.14 |
| Galactose metabolism | 0.4527 | 0.04 | Glutathione metabolism | 0.5881 | 0.26 |
| Purine metabolism | 0.5252 | 0.17 | Pentose phosphate pathway | 0.9407 | 0.21 |
| **Mid** | | | | | |
| **CTRL/ PBS+GnIH** | **p-value** | **Impact** | **CTRL/ GnIH+GnIH** | **p-value** | **Impact** |
| Gly, Ser and Thr metabolism | 0.0502 | 0 | Pantothenate and CoA biosynthesis | 0.0084 | 0 |
| Arginine and proline metabolism | 0.0695 | 0.10 | Aminoacyl-tRNA biosynthesis | 0.0121 | 0 |
| Ala, Asp and Glu metabolism | 0.1954 | 0.36 | Ala, Asp and Glu metabolism | 0.0124 | 0.36 |
| Aminoacyl-tRNA biosynthesis | 0.2608 | 0 | Purine metabolism | 0.0133 | 0.17 |
| Pyrimidine metabolism | 0.3572 | 0.02 | Gln and Glu metabolism | 0.0216 | 0.5 |
| Gln and Glu metabolism | 0.3617 | 0.5 | Arginine biosynthesis | 0.0216 | 0.12 |
| Arginine biosynthesis | 0.3617 | 0.12 | Glyoxal and Dicarbox metabolism | 0.0216 | 0 |
| Glyoxal and Dicarbox metabolism | 0.3617 | 0 | Nitrogen metabolism | 0.0216 | 0 |
| Nitrogen metabolism | 0.3617 | 0 | Pyrimidine metabolism | 0.0230 | 0.02 |
| Pantothenate and CoA biosynthesis | 0.5322 | 0 | Starch and sucrose metabolism | 0.0852 | 0.53 |
| Starch and sucrose metabolism | 0.5782 | 0.53 | Gly, Ser and Thr metabolism | 0.1918 | 0 |
| Purine metabolism | 0.6246 | 0.17 | Arginine and proline metabolism | 0.2354 | 0.1 |
| **Late** | | | | | |
| **CTRL/ PBS+GnIH** | **p-value** | **Impact** | **CTRL/ GnIH+GnIH** | **p-value** | **Impact** |
| Histidine metabolism | 0.0287 | 0 | Pyrimidine metabolism | 0.0061 | 0.02 |
| beta-Alanine metabolism | 0.0287 | 0 | Sphingolipid metabolism | 0.0078 | 0.01 |
| Purine metabolism | 0.0307 | 0.05 | TCA cycle | 0.0083 | 0.06 |
| Pyrimidine metabolism | 0.0446 | 0.02 | Glycerophospholipid metabolism | 0.0101 | 0.04 |
| Arginine biosynthesis | 0.0461 | 0 | Butanoate metabolism | 0.0108 | 0 |
| Propanoate metabolism | 0.1462 | 0 | Tyrosine metabolism | 0.0166 | 0.02 |
| Biosynthesis of unsaturated fatty acids | 0.1842 | 0 | Pyruvate metabolism | 0.0166 | 0 |
| Butanoate metabolism | 0.2504 | 0 | Biosynthesis of unsaturated fatty acids | 0.0186 | 0 |
| Ketone bodies metabolism | 0.2841 | 0 | Propanoate metabolism | 0.0277 | 0 |
| TCA cycle | 0.5722 | 0.06 | Ketone bodies metabolism | 0.0279 | 0 |
| Glycerophospholipid metabolism | 0.7757 | 0.04 | Arginine biosynthesis | 0.0356 | 0 |
| Tyrosine metabolism | 0.7836 | 0.02 | Gly, Ser and Thr metabolism | 0.0496 | 0.19 |
| Pyruvate metabolism | 0.7836 | 0 | Glyoxal and Dicarbox metabolism | 0.0496 | 0.04 |
| Gly, Ser and Thr metabolism | 0.8491 | 0.19 | Cys and Met metabolism | 0.0496 | 0.02 |
| Glyoxal and Dicarbox metabolism | 0.8491 | 0.04 | Purine metabolism | 0.2047 | 0.05 |
| Cys and Met metabolism | 0.8491 | 0.02 | Histidine metabolism | 0.4272 | 0 |
| Sphingolipid metabolism | 0.9411 | 0.01 | beta-Alanine metabolism | 0.4272 | 0 |
